# The transcriptional landscape and dynamics regulating organ differentiation and dormancy in *Curcuma alismatifolia*

**DOI:** 10.1093/plphys/kiaf501

**Published:** 2025-10-13

**Authors:** Xuezhu Liao, Mengmeng Hou, Yixuan Liu, Bing Xu, Xiaolong Huang, Christophe Bailly, Minlong Jia, Tengbo Huang, Zhiqiang Wu

**Affiliations:** Guangdong Provincial Key Laboratory for Plant Epigenetics, College of Life Sciences and Oceanography, Shenzhen University, Shenzhen 518060, China; State Key Laboratory of Tropical Crop Breeding, Shenzhen Branch, Guangdong Laboratory of Lingnan Modern Agriculture, Key Laboratory of Synthetic Biology, Ministry of Agriculture and Rural Affairs, Agricultural Genomics Institute at Shenzhen, Chinese Academy of Agricultural Sciences, Shenzhen 518120, China; School of Breeding and Multiplication (Sanya Institute of Breeding and Multiplication), Hainan University, Sanya 572000, China; State Key Laboratory of Tropical Crop Breeding, Shenzhen Branch, Guangdong Laboratory of Lingnan Modern Agriculture, Key Laboratory of Synthetic Biology, Ministry of Agriculture and Rural Affairs, Agricultural Genomics Institute at Shenzhen, Chinese Academy of Agricultural Sciences, Shenzhen 518120, China; Guangxi Key Laboratory for Polysaccharide Materials and Modifications, Guangxi Minzu University, Nanning 530006, China; State Key Laboratory of Tropical Crop Breeding, Shenzhen Branch, Guangdong Laboratory of Lingnan Modern Agriculture, Key Laboratory of Synthetic Biology, Ministry of Agriculture and Rural Affairs, Agricultural Genomics Institute at Shenzhen, Chinese Academy of Agricultural Sciences, Shenzhen 518120, China; College of Horticulture, Shanxi Agricultural University, Taiyuan 030031, China; Guangdong Provincial Key Laboratory for Plant Epigenetics, College of Life Sciences and Oceanography, Shenzhen University, Shenzhen 518060, China; Laboratoire de Biologie du Développement, Sorbonne Université, CNRS, Paris 75252, France; State Key Laboratory of Tropical Crop Breeding, Shenzhen Branch, Guangdong Laboratory of Lingnan Modern Agriculture, Key Laboratory of Synthetic Biology, Ministry of Agriculture and Rural Affairs, Agricultural Genomics Institute at Shenzhen, Chinese Academy of Agricultural Sciences, Shenzhen 518120, China; College of Horticulture, Shanxi Agricultural University, Taiyuan 030031, China; Guangdong Provincial Key Laboratory for Plant Epigenetics, College of Life Sciences and Oceanography, Shenzhen University, Shenzhen 518060, China; State Key Laboratory of Tropical Crop Breeding, Shenzhen Branch, Guangdong Laboratory of Lingnan Modern Agriculture, Key Laboratory of Synthetic Biology, Ministry of Agriculture and Rural Affairs, Agricultural Genomics Institute at Shenzhen, Chinese Academy of Agricultural Sciences, Shenzhen 518120, China; College of Horticulture, Shanxi Agricultural University, Taiyuan 030031, China

## Abstract

The emergence of specialized organs represents key evolutionary innovations that enable plants to thrive in diverse environments. However, the developmental mechanisms underlying these traits, particularly those of underground storage organs like rhizomes, remain poorly understood. Siam tulip (*Curcuma alismatifolia* Gagnep.), with its unique suite of modified organs (such as bracts, rhizomes, and tuberous roots) and dual reproductive strategies through seeds and rhizomes, serves as an ideal model for exploring organ differentiation and dormancy regulation. Through a comprehensive organ-wide transcriptomic analysis, we revealed functional differentiation and conservation across *C. alismatifolia* organs. For example, the outer bracts retain photosynthetic capacity similar to leaves, while the inner bracts have lost this function. The rhizome, a critical reproductive organ, acts as both a nutrient reservoir and a dormancy-driven survival mechanism in adverse conditions. Using Weighted Gene Co-expression Network Analysis, we identified transcription factors (TFs) associated with ABRE *cis*-acting elements as key regulators of rhizome development. By integrating transcriptomic data with high-temperature and phytohormone treatments, heterologous expression, dual-luciferase reporter assays and yeast 1-hybrid assays, we demonstrated the central role of cytochrome *P450* (*P450*) genes, particularly *ABA 8′-hydroxylase 1* (*CYP707A1*), in regulating rhizome dormancy release and high-temperature responses. Moreover, we showed that *CYP707A1* is regulated by the MYB TF 96 (MYB96), WRKY TF 35 (WRKY35), AP2/ERF and B3 domain-containing TF RAV1 (RAV1), and Two-component response regulator ARR18 (ARR18) TFs, offering potential strategies for year-round production. This study establishes *C. alismatifolia* as a powerful model for investigating the formation and specialization of evolutionary innovations like rhizomes and bracts, highlighting their adaptive mechanisms and resilience to environmental challenges in Zingiberaceae.

## Teaser

Insights into the genetic regulation of organ specialization and rhizome dormancy in Siam tulip advance understanding of how underground organs support plant survival and agricultural use.

## Introduction

The colonization of land by multicellular plants around 500 million years ago was a defining evolutionary breakthrough, transforming Earth's ecosystems, atmosphere, and carbon cycle. This success was driven by key structural innovations—roots, vascular systems, and seeds—that allowed plants to adapt and thrive in terrestrial environments (Weng et al. 2021; Preston et al. 2022). Over time, plants evolved specialized organs, offering novel solutions to environmental challenges, such as rhizomes and tubers, which enable survival by storing nutrients like starch and secondary metabolites. These organs not only provide energy reserves for enduring harsh conditions but also delay sprouting to synchronize growth with favorable environmental cues (Murthy et al. 2024). For example, the potato (*Solanum tuberosum* L.) tuber serves as both a nutrient reservoir and asexual reproductive organ, enhancing resilience in adverse environments (Destefano-Beltrán et al. 2006a, 2006b; Zheng et al. 2012). Therefore, understanding the developmental mechanisms underlying different organs is crucial for unlocking their full potential, paving the way for targeted improvements and innovations in plant adaptation and productivity.

**Figure 1.**
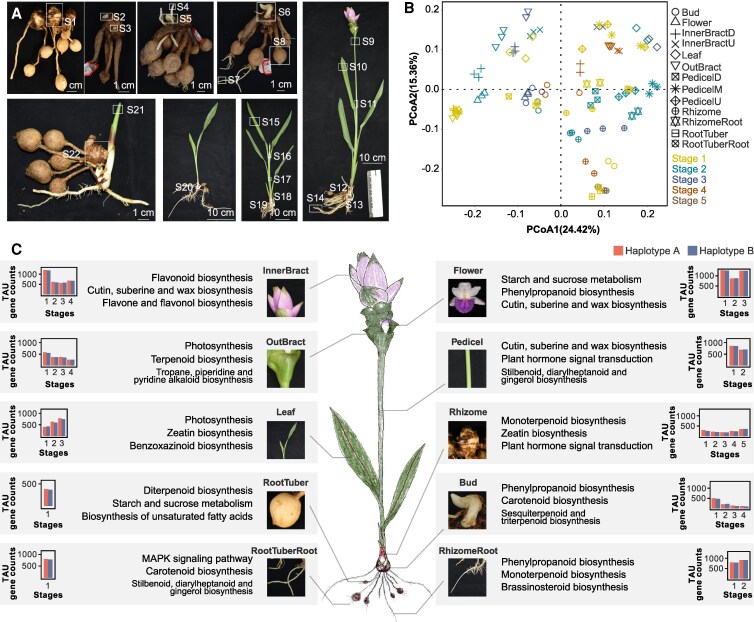
Organ specificity in *C. alismatifolia*. **A)** Spatial distribution of samples collected for transcriptome analysis (see Materials and Methods for sampling details; complete sample information in Supplementary Table S1, D/M/U represent the base, middle, and tip of the sampling organ, respectively.). **B)** PCoA analysis of RNA-seq data derived from all 13 organ types across distinct developmental stages. **C)** Identification of organ-preferential genes across organs and developmental stages, with functional characterization based on GO and KEGG enrichment analyses. Bar graphs indicate the number of organ-preferential expressed genes (*y*-axis) within each organ at progressively later developmental stages (*x*-axis; see Supplementary Table S1 for stage correspondence). HapA, Haplotype A-preferentially expressed genes shown in red; HapB, Haplotype B-preferentially expressed genes shown in blue. Except for the flower image, the remaining images are identical to those in Fig. 1A.

*Curcuma*, a cornerstone of the Zingiberaceae family (Gui et al. 2020; Liang et al. 2022; Yang et al. 2023), exemplifies evolutionary innovation through its diverse specialized organs, such as aromatic rhizomes, modified bracts, and tuberous roots (Wu 2012). Thriving across subtropical and tropical Asia, this family is celebrated not only for its culinary, medicinal, and ecological importance but also for its unique reproductive strategies and specialized pollination mechanisms (Wang et al. 2004; Sun et al. 2011). Among its members, Siam tulip (*Curcuma alismatifolia* Gagnep.), is a notable representative of Zingiberaceae with hallmark modified organs, including vibrant bracts, rhizomes, and tuberous roots (Fig. 1A). Its vividly colored and uniquely shaped bracts make it a rising star in the horticultural industry, widely cultivated as a popular cut flower and potted ornamental plant in China and Southeast Asia (Fukai and Udomdee 2005; Ye et al. 2007; Taheri et al. 2012, 2014; Lihui et al. 2018). Its rhizomes, used for asexual reproduction, such as those of ginger (*Zingiber officinale* Roscoe) and turmeric (*Curcuma longa* L.), play a vital role in adaptation, storing carbohydrates to fuel overwintering and rapid sprouting in the next season (Panneerselvam et al. 2007; Chen et al. 2020). These rhizomes not only support agricultural yields in *C. alismatifolia* similar to ginger and lotus but also house potential valuable secondary metabolites prized for their antioxidant and antimicrobial properties (Yang et al. 2015; Yoshida et al. 2016; Kochaphum et al. 2019; Chen et al. 2020; Guo et al. 2021). These compounds are a testament to the dual brilliance of nature—ensuring survival and delivering economic value. However, rhizome dormancy, a universal characteristic of the Zingiberaceae (Panneerselvam et al. 2007; Thohirah et al. 2010; Wu 2012; Saljuna et al. 2023), presents a considerable challenge to its year-round production (Paisooksantivatana et al. 2001; Thohirah et al. 2010; Taheri et al. 2012), underscoring the need for improved dormancy regulation strategies to fully unlock its economic and ornamental potential. Overall, *C. alismatifolia* serves as an exceptional model for studying the evolution and functional specialization of plant organs, which can provide insights into adaptive innovations that bridge ornamental, ecological, and medicinal value.

Rhizome dormancy is a vital adaptive strategy that enables plants to tolerate unfavorable conditions as well as ensure survival and efficient resource utilization (Lian et al. 2024). While rhizome dormancy remains poorly understood, extensive research on seed dormancy provides a strong foundation for exploring similar mechanisms. In seeds, abscisic acid (ABA) and gibberellins (GAs) are central regulators of dormancy and germination. ABA, in particular, governs stress responses and developmental processes through a dynamic balance between synthesis and degradation, with ABA 8′-hydroxylase (*CYP707A*) playing a key role in its inactivation (Chono et al. 2013). For instance, *AtCYP707A* in *Arabidopsis* (*Arabidopsis thaliana* (L.) Heynh.) and *OsABA8ox1* in rice (*Oryza sativa* L.) control dormancy release by reducing ABA levels (Wang et al. 2016; Fu et al. 2022). Similar mechanisms are observed in tubers, where endogenous ABA is essential for initiating and maintaining dormancy in potato tubers. Disruption of *StCYP707A1* significantly increases ABA levels in dormant buds, prolonging dormancy (Destefano-Beltrán et al. 2006a, 2006b). Additionally, transcription factors (TFs), such as MADS-box, WRKY, NAC, TCP, and AP2/ERF families, are known to regulate organ development and dormancy in other systems. For example, StTCP15 in potato balances ABA and GAs to regulate tuber sprouting, while NAC and MYB TFs influence cellulose accumulation in ginger rhizomes as ABA and cytokinin levels decline (Hu et al. 2011; Yang et al. 2015; Liu et al. 2018; Alvarez-Buylla et al. 2019; Chen et al. 2020; Hou et al. 2020; Gao et al. 2023; Nelson et al. 2023; Wu et al. 2023). While quantitative trait loci related to rhizome development have been identified and large-scale transcriptomic and proteomic analyses have revealed key pathways (Yoshida et al. 2016; Lian et al. 2024), the essential genes regulating rhizome development and dormancy in Zingiberaceae remain elusive. Given the parallels between seeds, tubers, and rhizomes, these existing studies provide valuable clues and a roadmap for understanding rhizome dormancy and development in Zingiberaceae.

This study presents a comprehensive organ-wide transcriptome of *C. alismatifolia*, revealing distinct functional specialization in its modified organs, such as bracts, rhizomes, and tuberous roots. In particular, key TFs linked to ABA-responsive elements (ABRE) *cis*-acting elements and *CYP707A1* genes were identified as pivotal regulators of rhizome development, dormancy release, and heat responses in *C. alismatifolia*. These findings enhance our understanding of innovative plant traits like rhizomes, which are crucial for survival and adaptation. By uncovering the mechanisms behind rhizome dormancy and organ differentiation, this work provides candidate genes for year-round production of *C. alismatifolia* while offering strategies to improve economically important crops such as ginger and turmeric.

## Results

### Unveiling the function of metamorphic organs through comprehensive whole-organ transcriptional profiles construction

To assess the functions performed by each organ of *C. alismatifolia* and to compare the shared and divergent functions between modified and ancestral organs, we calculated the tissue specificity index (TAU) of all genes to determine their organ-preferential expression based on our previously published transcriptome data obtained from various organs and developmental stages (Liao et al. 2022, 2024; Fig. 1, A and B, Supplementary Fig. S1 and Tables S1 and S2). Genes with TAU ≥ 0.8 were considered organ-preferential. During the early stages of organ development, higher gene expression levels were observed in organs such as buds, florets, bracts, and rhizomes, reflecting the involvement of more genes in early organogenesis (Fig. 1C). Furthermore, genes in certain organs, such as rhizomes, tuberous roots, and early-stage buds, display similar expression patterns due to their shared subsurface characteristics and functions (Fig. 1B, Supplementary Fig. S2). Functional enrichment of organ-preferential expressed genes revealed that genes preferentially expressed in upper inner bracts were enriched in flavonoid biosynthesis pathways, consistent with their anthocyanin accumulation. Interestingly, photosynthesis-related genes were enriched in outer bracts and leaves but not in inner bracts. This aligns with phenotypic observations, where inner bracts focus on displaying vivid colors while outer bracts retain photosynthetic functions. However, some overlapping traits were noted, for example, chlorophyll accumulation was observed at the tips of inner bracts, and small amounts of anthocyanin was found in outer bracts (Fig. 1, A and C). These findings suggest differentiation between bracts and leaves, with lower bracts representing a transitional state, displaying characteristics of both bracts and leaves.

In underground organs, such as rhizomes, roots, and tuberous roots, functional enrichment analyses indicated responsiveness to stimuli. Genes involved in starch and sugar metabolism were significantly enriched in tuberous roots (*P* < 0.05, hypergeometric test), aligning with their function as specialized nutrient storage organs (Fig. 1C, Supplementary Tables S3 and S4). Starch staining experiments in rhizomes and tuberous roots of different *C. alismatifolia* cultivars revealed starch accumulation in both, but with distinct coloration patterns, suggesting differences in starch composition. Amylose and amylopectin form complexes with iodine, appearing blue and red-purple, respectively (Han et al. 2019 ). Tuberous roots showed a blue reaction, indicating higher amylose content, whereas rhizomes displayed blue-purple, further supporting the role of rhizomes as both reproductive and storage organs. Even in the absence of tuberous roots, rhizomes can support vegetative propagation (Fig. 1C, Supplementary Fig. S3A). Additionally, targeted metabolomic analysis of rhizomes at harvest revealed terpenoids, phenolic acids, flavonoids, and lipids as major secondary metabolites, with terpenoids being the most abundant, consistent with the enrichment of terpene biosynthesis pathways in rhizomes and highlight the potential medicinal value of *C. alismatifolia* rhizomes (Fig. 1C, Supplementary Fig. S3B).

Using a stricter threshold (TAU ≥ 0.99) to identify genes with stronger organ specificity, we identified 520 genes (Haplotype A: 270, Haplotype B: 249, Supplementary Fig. S4 and Table S5). Functional annotation revealed well-known organ-specific genes, such as AGAMOUS-like MADS-box protein (*AGL*) and SEPALLATA (*SEP*) genes involved in floral development, which were specifically expressed in floral organs. Additionally, MYB and MYB-like TFs were specifically expressed in florets and tuberous roots, suggesting their roles in regulating organ-specific development. These organ-specific genes also provide candidate marker genes for future single-cell sequencing studies to explore earlier stages of organogenesis and development (Supplementary Fig. S4). Furthermore, leveraging 37,228 colinear gene pairs between 2 haplotypes previously identified through synteny analysis within our haplotype-resolved *C. alismatifolia* genome (Liao et al. 2024), we assessed their expression bias. The analysis revealed organ-specific expression divergence in only 100 gene pairs, indicating minimal functional divergence between alleles in this species (Supplementary Fig. S5 and Table S6).

### ABRE-related TFs involved in rhizome development

**Figure 2.**
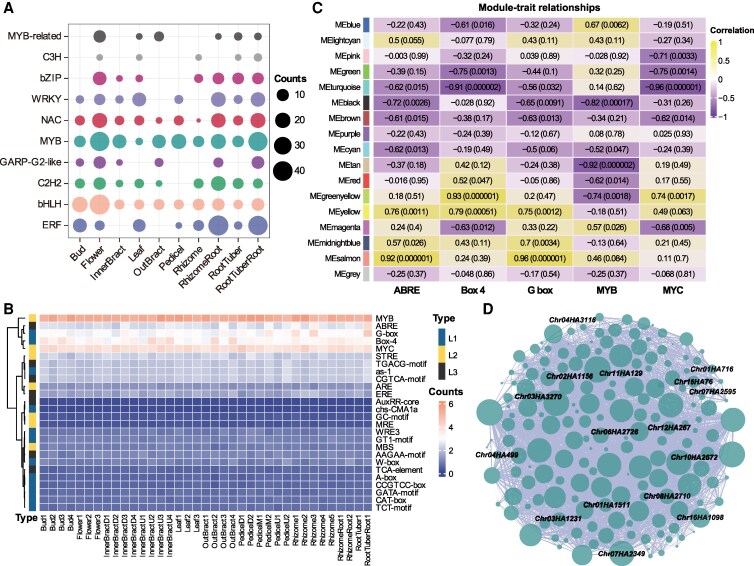
ABRE-related TFs in early rhizome development of *C. alismatifolia*. **A)** Distribution of the number of organ-preferentially expressed TFs (top 10 families) across both haplotypes. **B)** Mean density of *cis*-acting elements in 2 kb promoter regions of organ-preferentially genes from both haplotypes. L1, plant growth and development-related *cis*-acting elements (blue); L2, stress responsive *cis*-acting elements (yellow); L3, phytohormone responsive *cis*-acting elements (black). **C)** WGCNA heatmap showing correlation between early-expressed genes and their 5 major *cis*-acting elements (analyzed using only Haplotype A to minimize repeated gene interference). **D)** Connection network of hub genes in salmon module positively correlated with the ABRE *cis*-acting elements, in which TFs are labeled. The size of the circle indicates the connectivity, while the thickness of the line indicates the weight.

To investigate which TFs may be involved in the formation and development of specialized organs in *C. alismatifolia*, we identified TFs and filtered for the top 10 categories with the highest number of organ-preferentially expressed genes. Among these, the largest categories were MYB (Haplotype A: 99, Haplotype B: 100) and bHLH (Haplotype A: 71, Haplotype B: 74), followed by AP2/ERF (Haplotype A: 74, Haplotype B: 70) (Supplementary Tables S7 and S8). However, the organ preferences of these TFs varied. For instance, MYB, bHLH, NAC, and bZIP TFs were predominantly expressed in flowers, whereas ERF and WRKY TFs were more preferentially expressed in roots. In rhizomes, MYB, bHLH, ERF, and C2H2 TFs showed higher preferential expression, suggesting that the formation of different organs is shaped by the differential expression of diverse TFs (Fig. 2A).

Next, we analyzed the *cis*-acting elements within the 2 kb promoter regions of organ-preferentially expressed genes across different developmental stages. We observed a high abundance of MYB, MYC, Box 4, ABRE, and G-box elements, particularly MYB and MYC, which were present in nearly all promoter regions of preferentially expressed genes (Fig. 2B). This aligns with the dominance of MYB and bHLH TFs in these organs (Fig. 2A). Additionally, we identified a higher prevalence of ABRE and G-box elements in rhizomes and roots, which involved in ABA responsiveness, indicating the potential involvement of ABA-related genes in the formation and development of these organs (Fig. 2B, Supplementary Table S9).

To further validate key candidate TFs involved in organ development, we performed Weighted Gene Co-expression Network Analysis (WGCNA) using early-stage expression data from buds, flowers, leaves, outer bracts, rhizomes, and roots on rhizome. This analysis correlated gene expression modules with the abundance of the top 5 *cis*-acting elements from preferentially expressed genes in these samples (Fig. 2C, Supplementary Figs. S6 to S8). A salmon module associated with ABRE *cis*-acting elements was identified, showing a strong correlation between gene significance and module membership (Fig. 2C, Supplementary Fig. S9). Gene expression within the salmon module was markedly higher in rhizomes compared with other organs, further supporting the involvement of ABA-related genes in rhizome development (Supplementary Fig. S9B and Table S10). Within the salmon module, we identified 181 hub genes, including 16 TFs (Fig. 2D, Supplementary Table S11). Of these, 11 have characterized homologs in rice (*O. sativa* L.) or *Arabidopsis* (*A. thaliana* (L.) Heynh.) (7 of which respond to phytohormones such as ABA, GAs, BRs, auxin, and jasmonic acid [JA]), and 4 have been directly implicated in ABA signaling (Supplementary Table S11). Among these genes, 8 of which were preferentially expressed in rhizomes (Fig. 2D, Supplementary Table S11). Two TFs were highly expressed in early-stage rhizomes (Rhizome1) and are associated with ABA (Fig. 2D, Supplementary Fig. S10 and Table S11). One, Chr11HA129, was identified as a homolog of OsNAC52, a rice NAC TF known to respond to ABA and enhance drought tolerance in transgenic plants (Gao et al. 2010). Another gene, *Chr07HA2349*, was identified as a homolog of wheat (*Triticum aestivum* L.) jasmonate ZIM domain-containing protein gene (*TaTIFY10A)*, whose expression is induced by ABA and JA (Liu et al. 2024). These findings suggest that ABA-related TFs are involved in regulating rhizome development. TaTIFY10A, in particular, functions as a negative regulator of seed dormancy and plays an essential role in seed germination and abiotic stress responses in transgenic *Arabidopsis* and rice (Liu et al. 2024). In our study, we observed a marked downregulation of *Chr07HA2349* during rhizome development, with almost no expression in dormant rhizomes, suggesting that this TF participates in ABA-mediated regulation of rhizome development in *C. alismatifolia*.

### Involvement of the cytochrome *P450* (*P450*) superfamily in rhizome development

**Figure 3.**
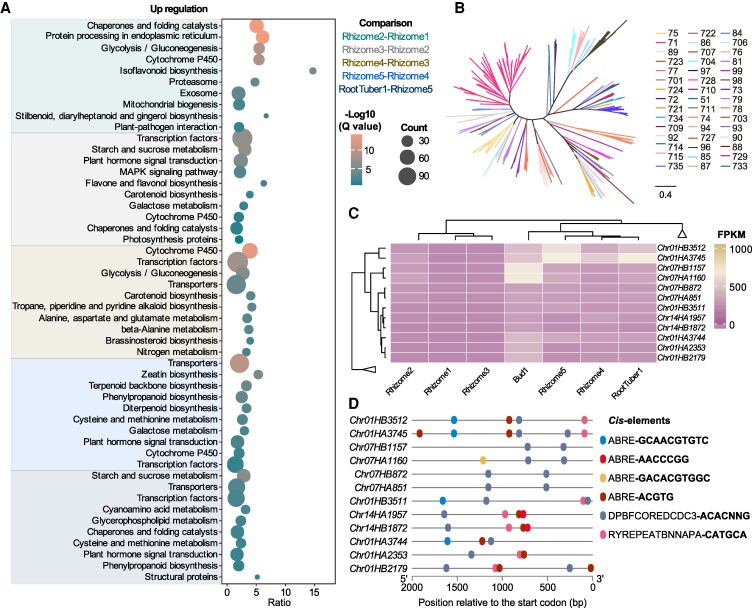
The cytochrome *P450* superfamily has been involved in rhizome development. **A)** KEGG enrichment of DEGs of Haplotype A during rhizome development and between rhizomes and tuberous roots in *C. alismatifolia*. **B)** Identification of cytochrome *P450* superfamily members and their phylogenetic relationship construction in *C. alismatifolia* and cytochrome *P450* superfamily members in rice as references. The scale bar indicates the number of amino acid substitutions occurring along the branch. A value of 0.4 means that, on average, 40 substitutions have occurred per 100 sites between sequences. **C)** Expression clustering of cytochrome *P450* superfamily members associated with rhizome dormancy and other genes are collapsed. **D)** ABA-related *cis*-acting elements in 2 kb promoter region of cytochrome *P450* superfamily members.

To further identify additional key candidate genes involved in rhizome development in *C. alismatifolia*, we performed Kyoto Encyclopedia of Genes and Genomes (KEGG) enrichment analysis on differentially expressed genes (DEGs) across various rhizome developmental stages. Early-stage DEGs were enriched in pathways such as isoflavonoid biosynthesis, flavone and flavonol biosynthesis, and stilbenoid, diarylheptanoid and gingerol biosynthesis. Notably, genes related to starch and sucrose metabolism began to up-regulate during the leaf-expansion stage and started to decline by the full-bloom stage, indicating the accumulation of photosynthetic products in storage organs (Fig. 3A, Supplementary Figs. S11 and S12 and Table S12). Interestingly, when comparing sprouting (Rhizome5) and dormant rhizomes (Rhizome4), the sprouting rhizomes exhibited higher expression of terpene biosynthesis genes, suggesting their potential involvement in early bud development. In contrast, starch and sucrose metabolism-related genes were more highly expressed in tuberous roots than in rhizomes (Fig. 3A, Supplementary Table S12), which is consistent with the higher amylose content in tuberous roots.

Additionally, cytochrome *P450* superfamily genes were enriched throughout all rhizome developmental stages. We then identified all cytochrome *P450* genes in the *C. alismatifolia* genome, detecting 317 in Haplotype A and 270 in Haplotype B, with the *CYP71* family being the most abundant (Fig. 3B). Similar to the tandemly duplicated *TPS* genes (Liao et al. 2024), members of the large cytochrome *P450* family, which typically comprises hundreds of members in plants (Wan et al. 2025), were also distributed in tandem duplication across the genome (Supplementary Fig. S13 and Table S13). Expression clustering of all *P450* genes revealed 6 genes highly expressed in buds and rhizomes related to the sprouting stage, including 1 *CYP51* gene (*Chr07HA851/Chr07HB872*), 1 *CYP73* gene (*Chr14HA1957/Chr14HB1872*), 2 *CYP707A* genes (*Chr07HA1160/Chr07HB1157* and *Chr01HA2353/Chr01HB2179*), and 2 *CYP71* genes (*Chr01HA3744/Chr01HB3511* and *Chr01HA3745/Chr01HB3512*) (Fig. 3C). Among these, the ABA 8′-hydroxylase gene *CYP707A* has been considered a sprouting marker gene (Zhang et al. 2021). Promoter analysis of these genes using PlantCARE and PLACE software revealed the presence of ABA-related *cis*-acting elements in their promoter regions, suggesting their involvement in breaking rhizome dormancy in *C. alismatifolia* (Fig. 3D, Supplementary Table S14).

### The ABA 8′-hydroxylase gene *CYP707A* is involved in rhizome dormancy release in *C. alismatifolia*

**Figure 4.**
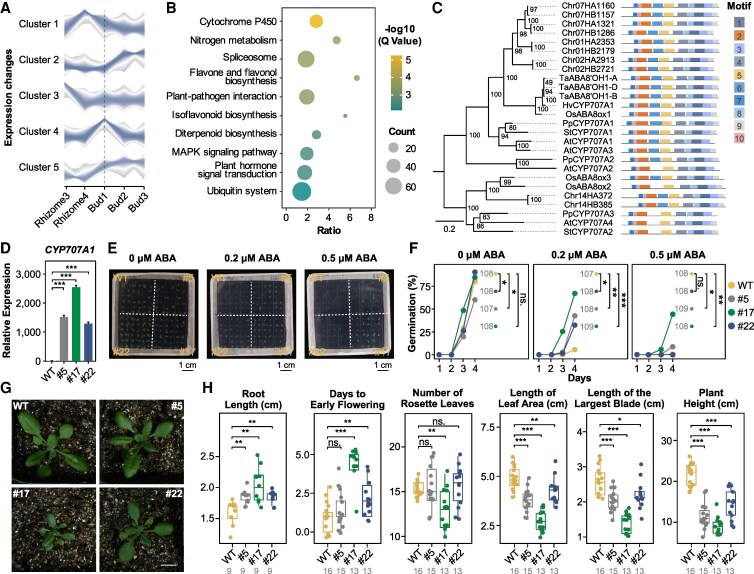
The ABA 8′-hydroxylase gene *CYP707A1* is involved in rhizome dormancy release in *C. alismatifolia*. **A)** Gene expression trends during dormancy release in *C. alismatifolia*. **B)** Functional enrichment of genes preferentially expressed in rhizomes and buds in Cluster 4. **C)** Phylogenetic tree and motifs of the *CYP707A1* gene. A value of 0.2 means that, on average, 20 substitutions have occurred per 100 sites between sequences. **D)** Validation of RT-qPCR for T2 lines of *CYP707A1* transgenic *A. thaliana*. Results are reported as mean ± standard deviation (Sd). *n* = 3. Statistical tests were 2-sided Student's *t*-test, and multiple comparisons were adjusted with the Bonferroni correction. Asterisks represented significant differences (***P < 0.001, adjusted). **E)** Seed germination of *CYP707A1*-overexpressing T2 lines on medium supplemented with varying ABA concentrations. **F)** Seed germination rate of transgenic lines. The dots in the figure represent the total germination rate of ∼108 seeds including seeds from 3 biological replicates. The gray numbers indicate the sample size. Statistical tests were 2-sided Student's *t*-test, and multiple comparisons were adjusted with the Bonferroni correction. Calculations were based on the germination rates observed on day 4 from 3 biological replicates (each replicate containing ∼36 seeds). Asterisks represented significant differences (*P < 0.05, **P < 0.01, ***P < 0.001, adjusted) (Supplementary Table S21). **G)** The phenotype of rosettes at bolting initiation in the transgenic lines (full phenotypes shown in Supplementary Fig. S17; For each image, bar = 1 cm). **H)** Quantitative traits of transgenic plants, including root length at 10 d post-germination, days to early flowering, number of rosette leaves at WT plants bolting, length of leaf area and maximum leaf blade length at WT plants bolting, and plant height at 50% flowering. The box-plot elements were defined as: center line, median; box limits, upper and lower quartiles; whiskers, 1.5× interquartile range; points, all values. Two-sided Wilcoxon rank-sum test was conducted for significance evaluation, and multiple comparisons were adjusted with the Bonferroni correction. Asterisks represented significant differences (*P < 0.05, **P < 0.01, ***P < 0.001, adjusted). WT, wild-type *Col-0*; #5, #17, #22: independent *CYP707A1* transgenic lines. The gray numbers indicate the sample size.

To further identify key genes regulating dormancy in *C. alismatifolia*, we conducted temporal trend analysis of DEGs during rhizome dormancy release using Mfuzz software. The analysis grouped these genes into 5 clusters, among which genes in Cluster 4 exhibited higher expression in sprouting buds (Bud1) compared with other samples (Fig. 4A, Supplementary Fig. S14). We further screened for genes preferentially expressed in rhizomes and buds within Cluster 4 and performed Gene Ontology (GO) and KEGG enrichment analysis (Supplementary Tables S15 and S16), and revealed hormone-related pathways, including cytochrome *P450*, diterpenoid biosynthesis, and plant hormone signal transduction enriched (Fig. 4B, Supplementary Tables S17 and S18). Notably, the previously mentioned *CYP707A* gene (*Chr07HA1160/Chr07HB1157*) was among the enriched cytochrome *P450* genes (Supplementary Table S18).

Then, we conducted a phylogenetic analysis of the *CYP707A* genes from *C. alismatifolia* alongside those from other species. The results showed that 4 *CYP707A* genes, including *Chr07HA1160* (its collinear gene on Haplotype B: *Chr07HB1157*), are closely related to *CYP707A1* genes associated with seed or bud dormancy and share conserved domains, such as *StCYP707A1* in potato (*S. tuberosum* L.), *PpCYP707A1* in peach (*Prunus persica* (L.) Batsch), *AtCYP707A1* and *AtCYP707A3* in *Arabidopsis* (*A. thaliana* (L.) Heynh.), *OsABA8ox1* in rice, and *CYP707A* genes in barley (*Hordeum vulgare* L.) and wheat (*T. aestivum* L.) (Fig. 4C). Functional studies show that knocking out *OsABA8ox1* in rice (*O. sativa* L.) significantly enhances seed dormancy (Fu et al. 2022), and *PpCYP707A1* in peach exhibits peak expression levels during the early stages of natural floral bud dormancy, suggesting a role in dormancy induction (Wang et al. 2016). Similarly, the knockout of *StCYP707A1* in potato induces higher ABA levels in dormant buds (Danieli et al. 2023). These findings suggest that the *CYP707A1* genes in *C. alismatifolia* may also regulate ABA levels and contribute to the release of rhizome dormancy.

Given that the *Chr07HA1160/Chr07HB1157* exhibit only a few amino acid differences and display similar expression trends in transcriptomic data (Fig. 3C, Supplementary Fig. S15), we selected the allele *Chr07HB1157* for heterologous expression in *A. thaliana* to validate the function of the *C. alismatifolia CYP707A1* gene. Combing PCR and RT-qPCR analysis identified expression levels of 3 transgenic *CYP707A1* lines were significantly higher in the T2 transgenic lines than in the wild *A. thaliana* (*P* < 0.001, 2-sided Student's *t*-test, Fig. 4D, Supplementary Fig. S16 and Tables S19 and S20). We further conducted seed germination experiments on T2 seeds from 3 transgenic lines (#5, #17, and #22). Seeds from wild-type (WT) *Col-0* and the transgenic lines were cultured on 1/2 MS medium containing different concentrations of ABA (0, 0.2, and 0.5 *μ*m), with daily monitoring of germination. The results showed that in the absence of ABA, germination rates for the transgenic lines surpassed those of the WT by Day 3, and the transgenic lines exhibited longer roots than the WT, indicating accelerated germination (Fig. 4, E and F). However, on high-ABA medium, germination rates of the WT and transgenic Lines #5 and #22 were inhibited, while Line #17 exhibited normal growth. Germination rates correlated with *CYP707A1* expression levels in each line (Fig. 4, D to F, Supplementary Table S21). Phenotypic analysis of the transgenic lines revealed that, as development progressed, the transgenic lines flowered significantly earlier than the WT (*P* < 0.05, 2-sided Wilcoxon rank-sum test, Fig. 4, G and H, Supplementary Fig. S17). At flowering, they displayed not only reduced height but also fewer and smaller rosette leaves, with Line #17 showing the most pronounced differences (Fig. 4, G and H, Supplementary Fig. S17). We therefore conclude that overexpression of *CYP707A1* not only accelerates seed germination but also affects subsequent vegetative growth and flowering-time traits. These findings demonstrate that *CYP707A1*, a key ABA catabolism gene from *C. alismatifolia*, plays a crucial role in breaking rhizome dormancy and influencing rhizome development.

### Rhizome dormancy is relieved in response to high temperature

**Figure 5.**
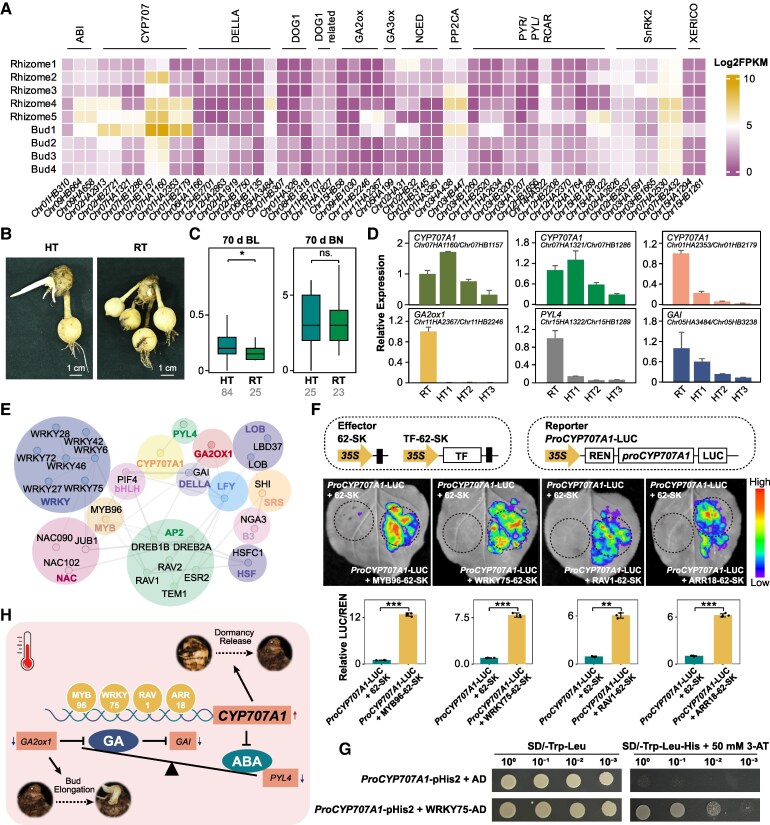
Rhizome dormancy release in response to high temperature in *C. alismatifolia*. **A)** Log_2_FPKM (Fragments Per Kilobase of exon model per Million mapped fragments) of genes associated with ABA and GAs in rhizomes and buds. **B)** Rhizome dormancy release at 70 d. Bar = 1 cm. RT, ambient conditions (22 ℃); HT, high temperature conditions (30 ℃). **C)** Number of bud and bud length of 70 d rhizomes. RT, ambient conditions (22 ℃); HT, high temperature conditions (30 ℃); BL, bud length; BN, number of bud. The gray numbers indicate the sample size. The box-plot elements were defined as: center line, median; box limits, upper and lower quartiles; whiskers, 1.5× interquartile range. Two-sided Wilcoxon rank-sum test was conducted for significance evaluation, and multiple comparisons were adjusted with the Bonferroni correction. Asterisks represented significant differences (**P < 0.05, adjusted) (Supplementary Tables S23 and S24). **D)** Expression of candidate genes in rhizomes at 70 d based on RT-qPCR. *n* = 3. RT, ungerminated buds in ambient conditions (22 ℃); HT1, ungerminated buds in high temperature conditions (30 ℃); HT2, 0.1 to 0.5 cm buds under high temperature conditions (30 ℃); HT3, 2 to 3 cm buds under high temperature conditions (30 ℃). Results are reported as mean ± Sd. **E)** Interaction network of TFs preferentially expressed during rhizome dormancy release with ABA-related candidate genes identified based on *Arabidopsis* homologs in the STRING database. **F)** Dual-luciferase reporter assays showed 4 candidate TFs (MYB96, WRKY35, RAV1, and ARR18) regulated the promoter activity of *CYP707A1* in the leaves of *N. benthamiana*. Relative LUC activity was normalized to REN activity (LUC/REN). Data represent the mean ± Sd (*n* = 3 to 4 biologically independent repeats. Statistical tests were 2-sided Student's *t*-test, and multiple comparisons were adjusted with the Bonferroni correction. Asterisks represented significant differences (**P < 0.01, ***P < 0.001, adjusted). Schematic diagrams of the reporter and effector constructs used in the dual-luciferase reporter assays. REN, Renilla luciferase; LUC, firefly luciferase. **G)** Y1H assay showed the WRKY75, which has the most predicted binding sites, bind to the *CYP707A1* promoter in yeast cells. **H)** Schematic diagram of rhizome dormancy release in *C. alismatifolia*. Increased expression of the *CYP707A1* gene promotes rhizome dormancy release, and decreased expression of the *GA2ox1* gene promotes bud elongation. Elevated temperature favored the rhizome dormancy release in *C. alismatifolia*. Black solid arrows indicate activation, black blunt-ended arrows indicate inhibition, red upward arrows indicate gene up-regulation, and dark blue downward arrows indicate gene down-regulation. Some rhizome images are the same as those in Fig. 1A.

ABA and GAs play crucial roles in dormancy release in bulbs (Zhao et al. 2022), we identified key GAs- and ABA-related genes based on functional annotation and BLAST analysis against *A. thaliana* homologs. These included ABA INSENSITIVE (ABI), ABA HYPERSENSITIVE GERMINATION 1 (AHG1), ABA 8′-hydroxylase (CYP707A), INDUCER OF CBF EXPRESSION (ICE1), 9-CIS-EPOXYCAROTENOID DIOXYGENASE (NCED), Protein phosphatase 2C clade A (PP2CA), PYRABACTIN RESISTANCE1 (PYR1)/PYR1-LIKE (PYL)/REGULATORY COMPONENTS OF ABA RECEPTORS (RCAR), SNF1-related protein kinase 2 (SnRK2), XERICO (XER), ASPARTIC ACID-GLUTAMIC ACID-LEUCINE-LEUCINE-ALANINE (DELLA), Gibberellin 20-Oxidase (GA20ox), Gibberellin 3-Oxidase (GA3ox), Gibberellin 2-Oxidase (GA2ox), and DELAY OF GERMINATION (DOG1). After excluding genes preferentially expressed in other organs, 6 candidate genes showing differential expression in sprouting states (Rhizome5 and Bud1) compared with the dormant state (Rhizome4) but not at other developmental stages were selected, including 3 *CYP707A1* genes (*Chr07HA1160/Chr07HB1157*, *Chr07HA1321/Chr07HB1286*, *Chr01HA2353/Chr01HB2179*), 1 *DELLA* gene *GAI* (*Chr05HA3484/Chr05HB3238*), 1 *GA2ox1* gene (*Chr11HA2367/Chr11HB2246*), and 1 *PYR/PYL/RCAR* gene *PYL4* (*Chr15HA1322/Chr15HB1289*) (Fig. 5A, Supplementary Table S22).

Given that temperatures of 30 °C favor rhizome dormancy release in *C. alismatifolia* (Paz et al. 2004) and that previous studies in potato tubers have shown that high-temperature treatment promotes tuber sprouting, accompanied by the activation of ABA signaling pathway genes (Zhang et al. 2021). To explore the effect of high temperatures on rhizome dormancy in *C. alismatifolia*, rhizomes were incubated at 30 °C (near-sprouting temperature) and 22 °C (control temperature). After 40 d, rhizomes at 30 °C exhibited longer lengths compared with the control, and this difference became more pronounced after 70 d, suggesting that high temperatures accelerate dormancy release (Fig. 5, B and C, Supplementary Tables S23 and S24). During this process, the expression of ABA degradation pathway genes (*CYP707A1*, *Chr07HA1160/Chr07HB1157*, and *Chr07HA1321/Chr07HB1286*) increased progressively in the transcriptome and was higher in ungerminated buds under high temperatures than in ungerminated buds at normal temperatures, and expression levels subsequently decreased in elongated buds, consistent with transcriptomic patterns observed during later bud development (Fig. 5, A and D, Supplementary Table S25). These findings reveal that *CYP707A1* genes promote ABA degradation primarily during the early stages of sprouting. Additionally, genes involved in the GAs signaling pathway (*GA2ox1*, *Chr11HA2367/Chr11HB2246*; *GAI*, *Chr05HA3484/Chr05HB3238*), the ABA receptor pathway (*PYL4 Chr15HA1322/Chr15HB1289*), and another *CYP707A1* gene (*Chr07HA1321/Chr07HB1286*) exhibited distinct changes during sprouting (Fig. 5D, Supplementary Table S25). These genes were highly expressed in ungerminated buds but down-regulated in sprouting buds, indicating their involvement in dormancy release. Notably, the expression pattern of *CYP707A1* (*Chr07HA1321/Chr07HB1286*) differed from that of *Chr07HA1160/Chr07HB1157*, suggesting a potential role as a negative regulator (Fig. 5D).

To further validate the function of GAs-related genes, we selected the *GA2ox1* gene *Chr11HA2367* as a candidate for heterologous expression in *A. thaliana*. The RT-qPCR analysis identified expression levels of 3 transgenic *GA2ox1* lines were significantly higher in transgenic lines compared with WT plants (*P* < 0.001, 2-sided Student's *t*-test) and correlated with plant height at 50% flowering, where transgenic lines, particularly Line #14, exhibited significantly reduced plant height compared with the WT (*P* < 0.05, 2-sided Wilcoxon rank-sum test, Supplementary Figs. S18 and S19). Seed germination experiments on T2 seeds showed no significant differences in germination rates between transgenic and WT lines, regardless of ABA presence (*P* > 0.05, 2-sided Student's *t*-test). These findings, combined with the expression changes observed during sprouting, suggest that *GA2ox1* primarily contributes to bud elongation after dormancy release (Supplementary Fig. S19, D and E and Table S21). Furthermore, TFs preferentially expressed during dormancy release were screened and analyzed for interactions with the candidate genes using the STRING database. Interaction network analysis based on evidence from *Arabidopsis* homologs suggests that TFs such as *MYB96, PIF4, AP2, NAC*, and *WRKY* may participate in rhizome dormancy release in *C. alismatifolia* (Fig. 5E, Supplementary Table S26). Building on predicted *cis*-regulatory elements within the 2 kb promoter region of *CYP707A1* (*Chr07HB1157*) and coherence in expression patterns, we identified 4 TFs: 3 predicted by the STRING database (MYB TF 96 [MYB96], WRKY TF 35 [WRKY35], and AP2/ERF and B3 domain-containing TF RAV1 [RAV1]) and an additional candidate (Two-component response regulator ARR18 [ARR18]) (Supplementary Fig. S20 and Table S27). Subsequent dual-luciferase reporter assays showed that these TFs regulated the promoter activity of *CYP707A1* in tobacco leaves (Fig. 5F), and further demonstrated that these 4 TFs could bind to the promoter of *CYP707A1* by yeast 1-hybrid (Y1H) assay (Fig. 5G, Supplementary Fig. S21). Based on these results, we propose a simplified model for dormancy release in *C. alismatifolia*, highlighting the role of high temperature in accelerating rhizome dormancy release through the modulation of ABA and GAs balance (Fig. 5H).

## Discussion

The evolution of land plants reshaped Earth, driving diversification and creating ecosystems that support terrestrial life. Unlike algae, land plants developed complex, specialized organs, which enhanced their adaptability. The origin of these morphological innovations remains a central challenge in evolutionary biology, as seen in carnivorous plants (Julca et al. 2021; Weng et al. 2021; Preston et al. 2022). In the Zingiberaceae family, *Curcuma* species often show overlapping and unclear morphological traits (Kress et al. 2002; Chen and Xia 2010; Liang et al. 2020). Siam tulip (*C. alismatifolia* Gagnep.), for instance, features unique modified organs such as bracts, rhizomes, and tuberous roots. To uncover how these specialized structures form and function, we analyzed the transcriptomes of various organs across developmental stages. Our results revealed principal components analysis (PCA) clustering aligned with phenotypic traits: outer bracts, leaves, and chlorophyll-containing inner bract tips grouped together, while the inner bracts are enriched in flavonoid biosynthesis pathways, the outer bracts retain photosynthetic genes found in leaves, suggesting they preserve photosynthetic function and act as an intermediary structure. Moreover, in *C. alismatifolia* cv. “Chiang Mai Pink,” fertile flowers are found in the green outer bracts, while the pink inner bracts primarily contain sterile flowers (Ding et al. 2021). Considering the terpene biosynthesis pathways enriched in the outer bracts (Fig. 1C), the high expression of terpene synthesis genes in outer bracts (Liao et al. 2024), and the signaling cascades triggered by volatile terpenes during reproductive organ development (Stirling et al. 2024), coupled with the well-established role of terpenes in plant internal communication and enhancing reproductive capacity (Boachon et al. 2019), we hypothesize that terpene compounds play a crucial role in the formation of fertile flowers in *C. alismatifolia*. Additionally, while both rhizomes and tuberous roots are key sites of starch synthesis, they serve distinct roles. Rhizomes function as organs of asexual reproduction, whereas tuberous roots are superior energy reservoirs. This study represents a step toward understanding the formation and functional differentiation of specialized plant organs through comprehensive transcriptomic analysis.

Rhizome dormancy is a key adaptive process that allows plants to survive in challenging conditions. ABA, a major regulator of dormancy, inhibits cell division and slows gene activity (Shu et al. 2016; Penfield 2017; Zhao et al. 2022), with its signaling involving PYR1/PYL/RCAR receptors, PP2CA phosphatases, and SnRK2 kinases (Nishimura et al. 2018). In our study, we observed increased expression of *PYL4* and *CYP707A1* during rhizome dormancy release, which then declined during bud development. *CYP707A1* remained highly expressed in sprouting buds, promoting ABA degradation, as confirmed by *Arabidopsis* (*A. thaliana* (L.) Heynh.) transgenics germinating under high ABA concentrations. Using WGCNA, we also innovatively linked ABA-responsive ABRE *cis*-acting elements to rhizome development and identified *TIFY10A* (*Chr07HA2349*) and *NAC52* (*Chr11HA129*) as down-regulated during rhizome development, with their homologs known to respond to ABA signaling (Gao et al. 2010; Liu et al. 2024). Additionally, high temperatures play a key role in dormancy release, as seen in potato (*S. tuberosum* L.) tuber sprouting (Zhao et al. 2022), where ABA signaling pathway genes are activated (Zhang et al. 2021). In *Arabidopsis*, high temperatures increase ABA levels in *Arabidopsis* seeds by activating ABA biosynthesis genes (e.g. *ABA1*, *NCED2,5,9*) and suppressing ABA degradation genes (e.g. *CYP707A1,2,3*) (Lim et al. 2013; Wang et al. 2021). However, in our study, high-temperature treatment resulted in longer buds and *CYP707A1* expression was higher in high-temperature dormant buds than in those under normal conditions but decreased after sprouting. This indicates that high temperatures promote *CYP707A1*-mediated ABA degradation, effectively breaking dormancy and enhancing sprouting.

For species like tulips and lilies, GAs break dormancy by promoting cell division, boosting energy metabolism, and reopening membrane communication (Li et al. 2019; Zhao et al. 2022). Key genes in GAs biosynthesis, such as *GA20ox*, *GA3ox*, and *GA2ox*, play crucial roles in this process (Han and Zhu 2011). In *Arabidopsis*, light inhibits *AtGA2ox2*, boosting gibberellin levels and promoting seed germination (Li et al. 2019 ). In our study, however, *GA2ox1* transgenic *Arabidopsis* plants did not release seed dormancy and exhibited late flowering, suggesting that *GA2ox1* does not play a primary role in rhizome dormancy release in *C. alismatifolia*. Moreover, high concentrations of GAs (100 to 600 mg/L) have been shown to delay germination in *C. alismatifolia* rhizomes (Khuankaew et al. 2009; Wang et al. 2021). Our findings align with this, as reducing GAs concentration to 20 mg/L had no significant effect on sprout number or length (*P* > 0.05, 2-sided Wilcoxon rank-sum test, Supplementary Fig. S22, A and B). After GAs treatment, *CYP707A1* expression in high-temperature non-germinating buds was slightly higher than in those at room temperature, with no down-regulation in elongating buds. Additionally, the down-regulation of *GA2ox1* in high-temperature non-germinating buds was suppressed (Supplementary Fig. S22C). These findings suggest that rhizome dormancy release in *C. alismatifolia* is likely linked to the ABA/GA balance, with high temperatures disrupting this balance, reducing ABA and increasing GAs, which promotes sprout germination and elongation. This complex process warrants further investigation, integrating seed and tuber dormancy studies, exploring the signaling networks involved, and uncovering the molecular mechanisms at play.

In summary, by constructing a comprehensive organ-wide transcriptome, we have explored the functional formation and differentiation of modified organs in *C. alismatifolia*, while identifying key genes that control rhizome dormancy release. This breakthrough lays a foundation for revolutionizing the year-round production of *C. alismatifolia* and other Zingiberaceae species, offering profound insights into their adaptive mechanisms of specialized organ development and driving innovations in the development and utilization of emerging ornamental crops.

## Materials and methods

### Acquisition of gene expression data

The haplotype genome, gene annotation files, and Fragments Per Kilobase of exon model per Million mapped fragments (FPKM) data for *C. alismatifolia* cv. “Chiang Mai Pink” were obtained from our previously generated datasets (Liao et al. 2022, 2024). RNA sequencing data were analyzed using STAR v2.7.10a (Dobin et al. 2013), with the merged haplotype genome of *C. alismatifolia* as the reference. Reads were mapped to the reference genome using the parameters --alignIntronMax 50000 --alignMatesGapMax 50000 --outFilterMismatchNmax 1 --outSAMattrIHstart 0, and read counts were calculated. FPKM values for genes on each haplotype were computed using StringTie v2.1.6. The RNA samples included bracts (QMF S1 Br [OutBract1], QMF S1 SeG [InnerBractU1], QMF S1 SeR [InnerBractD1], QMF S2 Br [OutBract2], QMF S2 SeG [InnerBractU2], QMF S2 SeR [InnerBractD2], QMF S3 Br [OutBract3], QMF S3 SeG [InnerBractU3], QMF S3 SeR [InnerBractD3], QMF S4 Br [OutBract4], QMF S4 SeG [InnerBractU4] [InnerBractD4], QMF S4 SeR [InnerBractD4]), florets (QMF KY1 [Flower1], QMF KY2 [Flower2], QMF KY3 [Flower3]), buds (0.1 to 0.5 cm Bud1, 0.5 to 1 cm Bud2, 1 to 2 cm Bud3, >2 cm Bud4), pedicels (the 2 cm upper pedicel [PedicelU1], the 2 cm middle pedicel [PedicelM1] and the 2 cm lower pedicel [PedicelD1] of flower initiation stage, the 2 cm upper pedicel [PedicelU2], the 2 cm middle pedicel [PedicelM2] and the 2 cm lower pedicel [PedicelD2] of blooming stage), rhizomes (Rhizome1 in the 2-leaf stage, Rhizome2 in flower initiation stage, Rhizome3 in blooming stage, and Rhizome4 at dormancy, Rhizome5 at bud emergence stage), tuberous root (RootTuber1 at Bud4), root on tuberous root at Bud4 (RootTuberRoot1), root on rhizomes at Bud3 (RootRhizomeRoot1), root on rhizomes at blooming stage (RootRhizomeRoot2), and the innermost whorl of about 10 cm Leaf (Leaf1), innermost leaf at flower initiation stage (Leaf2) and innermost leaf at blooming stage (Leaf3). Two to 3 biological replicates containing 3 plants each were performed for each sample (Liao et al. 2022, 2024). Complete sample metadata are provided in Supplementary Table S1. Based on the expression of all genes, corrplot v0.92 package (https://github.com/taiyun/corrplot) was used to calculate the correlation between samples. DESeq2 v1.38.3 (Love et al. 2014) was used to perform PCA analysis, and the vegan package (https://cran.r-project.org/web/packages/vegan/index.html) was used to perform principal co-ordinates analysis (PCoA).

### Identification and functional enrichment of organ-specific expressed genes

The tissue specificity index (TAU) has previously been used to identify organ-specific genes (Kryuchkova-Mostacci and Robinson-Rechavi 2017). Using the average FPKM of each sample, TAU values for each gene were calculated with the TAU_Calc tool in TBtools-II v2.136 (Chen et al. 2023) to determine the sample in which the gene is specifically expressed. Genes from both haplotypes with no expression across all samples were pre-filtered, and a threshold of TAU ≥ 0.8 was applied for further refinement. To identify organ-specific genes, TAU values were calculated based on the average FPKM of each organ, and genes were subsequently filtered with a stricter threshold of TAU ≥ 0.99. Functional annotation of the 520 identified organ-specific genes was performed using the NR database (https://ftp.ncbi.nlm.nih.gov/blast/db/FASTA/nr.gz). Additionally, GO and KEGG enrichment analysis was performed using TBtools-II v2.136 (Chen et al. 2023) based on the functional annotation results of the preferentially expressed genes from both haplotypes in eggNOG-Mapper v2.0.1 (Huerta-Cepas et al. 2017).

### TF and *cis*-acting element identification and WGCNA analysis

iTAK v1.7 (Zheng et al. 2016) was used to predict TFs in *C. alismatifolia*. Promoter regions of genes were extracted using TBtools-II v2.136 (Chen et al. 2023) based on the gene annotation file, and *cis*-acting elements in the extracted promoter sequences were identified using plantCARE (Lescot et al. 2002) and PLACE (Higo et al. 1999). The identified *cis*-acting elements were further classified into 3 categories: L1, plant growth and development-related *cis*-acting elements; L2, stress responsive *cis*-acting elements; L3, phytohormone responsive *cis*-acting elements (Mengarelli and Zanor 2021). To validate key candidate TFs involved in organ development, early expression data from buds, flowers, leaves, outer bracts, rhizomes, and roots on rhizome in Haplotype A (using only Haplotype A’s gene expression profiles to avoid redundant hub gene identification across haplotypes) were analyzed using a shiny app for WGCNA v0.0.6.230118 (Langfelder and Horvath 2008; Wang et al. 2024). Genes were filtered based on MAD values, resulting in 16,880 genes for further analysis. The minimum module size was set to 30. To obtain trait data required for WGCNA analysis and evaluate whether preferentially expressed genes were under the control of specific TFs, the preferentially expressed genes were filtered with a more stringent cutoff (TAU ≥ 0.8 and FPKM ≥ 1 in at least 1 sample) to reduce noise from low expression genes. Then, the average number of the top 5 *cis*-acting elements of these remaining preferentially expressed genes across samples was calculated, and their correlation with gene modules was assessed. Finally, candidate hub genes within the module were selected based on |KME| ≥ 0.5 and |GS| ≥ 0.5. The TFs among these hub genes were functionally annotated using the NR database (https://ftp.ncbi.nlm.nih.gov/blast/db/FASTA/nr.gz), and their interaction network was visualized with Cytoscape v3.10.3 (Shannon et al. 2003).

### Analysis of *P450* superfamily

BLAST+ v2.11.0 (https://ftp.ncbi.nlm.nih.gov/blast/executables/blast+/LATEST/) was used to identify candidate genes based on the *P450* superfamily members and their classifications in rice (*O. sativa* L.), as outlined by Wei and Chen (2018) . Sequence alignments were generated using MAFFT v7.490 (Katoh and Standley 2013), and poorly aligned regions were trimmed with TrimAL v1.4 (Capella-Gutierrez et al. 2009). A phylogenetic tree was constructed under the PROTCATWAG model using RAxML v8.2.12 (Stamatakis 2014). The chromosomal distribution of identified *P450* superfamily members was visualized with TBtools-II v2.136 (Chen et al. 2023).

To extract and classify *CYP707A* genes, amino acid sequences of *C. alismatifolia CYP707A* genes were aligned with those from other species using MAFFT v7.490 (Katoh and Standley 2013), including *AtCYP707A1* (At4g19230), *AtCYP707A2* (At2g29090), *AtCYP707A3* (At5g45340), and *AtCYP707A4* (At3g19270) from *Arabidopsis* (*A. thaliana* (L.) Heynh.), *HvCYP707A1* (BAF02839.1) from barley (*H. vulgare* L.), *OsABA8ox1* (LOC_Os02g47470.1), *OsABA8ox2* (LOC_Os08g36860.1), and *OsABA8ox3* (LOC_Os09g28390.1) from rice (*O. sativa* L.), *PpCYP707A1* (EMJ11776.1), *PpCYP707A2* (EMJ08520.1), and *PpCYP707A3* (EMJ02195.1) from peach (*P. persica* (L.) Batsch), *StCYP707A1* (ABA55732.1) and *StCYP707A2* (ABA55733.1) from potato (*S. tuberosum* L.), *TaABA8′OH1-A* (BAN28254.1), *TaABA8′OH1-B* (BAN28255.1), and *TaABA8′OH1-D* (BAN28256.1) from wheat (*T. aestivum* L.). Poorly aligned regions were trimmed with TrimAL v1.4 (Capella-Gutierrez et al. 2009), and a phylogenetic tree was constructed using IQ-TREE v2.0 (Minh et al. 2020) with 1,000 ultrafast bootstrap replicates to assess branch support. The phylogenetic tree was visualized using FigTree v1.4.3 (http://tree.bio.ed.ac.uk/software/Figtree).

### Identification of candidate genes for dormancy release in rhizome of *C. alismatifolia*

Gene expression trend analysis was conducted using FPKM data from Rhizome3, Rhizome4, Bud1, Bud2, and Bud3, with genes having FPKM values below 1 in all samples excluded. Clusters showing changes during dormancy were identified using the R package Mfuzz v2.58.0 (Kumar and Futschik 2007) and GO and KEGG functions were enriched for genes in Cluster 4. Based on TAU values, genes preferentially expressed in organs other than buds and rhizomes were removed from the candidate Cluster 4. Candidate genes involved in rhizome dormancy were further identified based on prior studies of GAs- and ABA-related genes (Schweighofer et al. 2004; Lim et al. 2013; Shu et al. 2016; Penfield 2017; Nishimura et al. 2018; Zhao et al. 2022) and annotations from eggNOG-Mapper v2.0.1 (Huerta-Cepas et al. 2017), including *ABI*, *AHG1*, *CYP707A*, *GA2ox*, *ICE1*, *NCED*, *PP2CA*, *PYR/PYL/RCAR*, *SnRK2*, *XERICO*, *DELLA*, *GA20ox*, *GA3ox*, and *DOG1*. Genes displaying expression patterns in Rhizome5 (dormancy release) and Bud1 (active growth) that differed from Rhizome4 (dormancy) were selected as final candidates. TFs were filtered under the same criteria. To construct functional protein association networks from the selected TFs and candidate genes, the amino acid sequences of all identified genes were submitted to the String v12.0 database (Szklarczyk et al. 2023) and the interaction networks were predicted based on homologous relationships with *A. thaliana* genes. To identify TFs regulating *CYP707A1*, we implemented a 3-tiered filtering strategy: (i) removal of TFs with FPKM ≤ 2 in any sample; (ii) exclusion of TFs lacking predicted binding sites in the *CYP707A1* promoter (*Chr07HB1157*; 2 kb upstream sequence), verified using JASPAR (https://jaspar.elixir.no/) and PlantPAN 4.0 (https://plantpan.itps.ncku.edu.tw/plantpan4/promoter_analysis.php); (iii) selection of TFs exhibiting expression patterns congruent with *CYP707A1* (i.e. elevated expression in Bud1 and Rhizome5) through Log_2_FPKM clustering of filtered candidates.

### Dual-LUC assay

The *CYP707A1* (*Chr07HB1157*) promoter was inserted into the pGreenII 0800-LUC vector to generate the reporter plasmid. Full-length coding sequences of 3 STRING-predicted candidates, MYB96 (Chr06HA2274), WRKY35 (Chr03HB3411), and RAV1 (Chr09HB325), along with an additional candidate (ARR18/Chr04HB516) based on expression pattern were inserted into the pGreenII 62-SK vector to create effector plasmids. All recombinant plasmids were transformed into *Agrobacterium tumefaciens* GV3101 (pSoup-p19). Transient expression assays were performed by co-infiltrating tobacco (*Nicotiana benthamiana*) leaves with *Agrobacterium* suspensions carrying reporter and effector constructs. Luminescence activity was quantified 60 h post-infiltration using a bioluminescence imaging system. Concurrently, infiltrated tobacco leaf samples were collected to detect luciferase activity using the Dual-Luciferase Reporter Assay Kit (Vazyme, China).

### Y1H assay

For WRKY35 (Chr03HB3411), the Y1H assay was conducted by cloning the *CYP707A1* promoter into the pHis vector. The coding sequences of the gene was inserted into the pGADT7 (AD) vector, respectively. The resulting construct was co-transformed with WRKY35-AD and an empty AD vector into yeast (*Saccharomyces cerevisiae*) cells. Selection was performed on SD/-Trp/-Leu/-His plates containing varying concentrations of 3-AT to determine the optimal 3-AT concentration for the assay. Validation of WRKY35 binding to *CYP707A1* promoter on SD/-Trp/-Leu/-His plates containing 50 mm 3-AT medium. For MYB96 (Chr06HA2274), RAV1 (Chr09HB325) and ARR18 (Chr04HB516), the CYP707A1 promoter was inserted into the pABAi vector, and validation of MYB96/RAV1/ARR18 binding to *CYP707A1* promoter were verified on SD/-Leu plates containing 5 mm 3-AT medium.

### Treatment of rhizomes

Dormant rhizomes (Rhizome4) were used for dormancy release experiments. The treatment group was soaked in 20 mg/L GAs for 6 h, while the control group was soaked in distilled water for the same duration. Half of the rhizomes from both groups were incubated at 22 °C, and the other half at 30 °C. Germination rates were recorded at 40 and 70 d. Each treatment included 3 biological replicates, with 7 to 9 rhizomes per replicate. For metabolic profiling, Rhizome4 samples were flash-frozen in liquid nitrogen and sent to Metware Biotechnology Inc. for widely targeted metabolomics analysis to identify and quantify secondary metabolites in the rhizomes.

To validate the expression of candidate genes following high-temperature and GAs treatments, total RNA was extracted from the treated rhizome samples using the EASYspin Plant RNA Kit (Aidlab, China). Reverse transcription was performed with the HiScript III 1st Strand cDNA Synthesis Kit (+gDNA wiper) (Vazyme, China). RT-qPCR was conducted using the ChamQ Universal SYBR qPCR Master Mix (Vazyme, China) using the CFX Connect Real-Time System (BIO-RAD, USA). Each gene was analyzed in triplicate, and relative expression levels were calculated through the comparative 2^−ΔΔ*CT*^ method. Primer sequences are listed in Supplementary Table S25.

### Generation of *CYP707A* and *GA2ox* transgenic *Arabidopsis* lines

The coding sequences of candidate genes *CYP707A1* (*Chr07HB1157*) and *GA2ox1* (*Chr11HA2367*) were extracted from the *C. alismatifolia* genome and the accuracy of the gene sequences and annotations was verified by aligning the previous reported genome and transcriptome sequencing data (Liao et al. 2024). The candidate genes were synthesized and inserted into the plant expression vector *p*CAMBIA1305 driving 35S promoter and transformed into *A. tumefaciens* GV3101. The *A. thaliana* ecotype Columbia *Col-0* was used as the WT. Transgenic *Arabidopsis* plants were generated using the floral dipping method, followed by a second transformation based on plant growth conditions. Seeds from T0 plants were collected and sown on 1/2 MS medium containing 50 *μ*g/mL hygromycin (Hyg). T1 seeds were harvested from individual plants, and this selection process was repeated to obtain homozygous T2 lines.

DNA extraction and PCR analysis were performed on T1 plants, and positive lines were retained for further analysis. Total RNA from T2 plants was extracted using the FastPure Universal Plant Total RNA Isolation Kit (Vazyme, China) and the cDNA was generated with the HiScript III 1st Strand cDNA Synthesis Kit (+gDNA wiper) (Vazyme, China). Diluted cDNA (10×) was used as the template for RT-qPCR, conducted with ChamQ Universal SYBR qPCR Master Mix (Vazyme, China) following the manufacturer's protocol, using *Arabidopsis Actin* as internal control (Supplementary Table S19). Reactions were run on a CFX Connect Real-Time System (BIO-RAD, USA). Each sample was analyzed in triplicate, and relative expression levels were calculated through the comparative 2^−ΔΔ*CT*^ method.

### Germination analysis of *Arabidopsis* seeds

Surface-sterilized seeds of *A. thaliana Col-0* WT and 3 transgenic lines of *Chr07HB1157* and *Chr11HA2367* (T2 generation) were sown on 1/2 MS medium supplemented with different concentrations of ABA (0, 0.2, and 0.5 *μ*m). The seeds were stratified at 4 °C for 48 h, then transferred to a growth chamber set at 22 °C under a 16-h light/8-h dark cycle. Each treatment included 3 biological replicates, with ∼36 seeds of WT and each positive transgenic line per replicate. Germination was defined as the emergence of cotyledons from the seed coat (Boyes et al. 2001). The number of germinated seeds was recorded daily for 1 to 4 d (Supplementary Table S21).

### Accession numbers

Sequence data from this article can be found in the Genome Sequence Archive at the National Genomics Data Center, Beijing Institute of Genomics, Chinese Academy of Sciences/China National Center for Bioinformation data libraries under accession number PRJCA017980 (https://ngdc.cncb.ac.cn/bioproject/browse/PRJCA017980) (Liao et al. 2024). All *C. alismatifolia* gene/protein sequences referenced in this study can be extracted from the *C. alismatifolia* genome assembly and annotation data on the Figshare (https://doi.org/10.6084/m9.figshare.25303411.v2) (Liao et al. 2024) using their respective IDs.

## Supplementary Material

kiaf501_Supplementary_Data

## Data Availability

No new data were generated or analyzed in support of this research.

## Supplementary data

The following materials are available in the online version of this article.

**Supplementary Figure S1.** Sample correlation analysis of *C. alismatifolia* RNA-seq data.

**Supplementary Figure S2.** PCA analysis of RNA-seq data derived from all 13 organ types across distinct developmental stages.

**Supplementary Figure S3.** Compounds from the *C. alismatifolia* rhizome.

**Supplementary Figure S4.** MADS box TFs specifically expressed in flowers.

**Supplementary Figure S5.** Colinear gene pairs with different preferred samples.

**Supplementary Figure S6.** Clustering of early samples across organizations.

**Supplementary Figure S7.** Analysis of network topology for various soft-thresholding powers.

**Supplementary Figure S8.** Module detection by WGCNA.

**Supplementary Figure S9.** Gene expression of salmon module associated with ABRE *cis*-elements.

**Supplementary Figure S10.** Expression of TFs in hub genes within the salmon module and preferentially expressed organs.

**Supplementary Figure S11.** Number of DEGs in different haplotypes during rhizome development.

**Supplementary Figure S12.** KEGG enrichment analysis of the down-regulated DEGs in Haplotype B during rhizome development and between rhizomes and tuberous roots in *C. alismatifolia*.

**Supplementary Figure S13.** Distribution of *P450* superfamily and terpene synthase (*TPS*) genes in both haplotypes of *C. alismatifolia*.

**Supplementary Figure S14.** Determination of the best cluster number for *k*-means.

**Supplementary Figure S15.** Coding sequence (CDS) alignment of the *CYP707A1* gene *Chr07HA1160/Chr07HB1157*.

**Supplementary Figure S16.** PCR validation of transgenic *A. thaliana* lines.

**Supplementary Figure S17.** Phenotypic characterization of *CYP707A1*-overexpressing *A. thaliana* plants.

**Supplementary Figure S18.** CDS alignment of the *GA2ox1* gene *Chr11HA2367/Chr11HB2246*.

**Supplementary Figure S19.** The T2 generation of *GA2ox*-overexpressing transgenic *A. thaliana* and its phenotypes.

**Supplementary Figure S20.** Expression clustering of candidate TFs that regulate *CYP707A1*.

**Supplementary Figure S21.** Y1H assay showing that MYB96, RAV1 and ARR18 bind to the *CYP707A1* promoter in yeast cells.

**Supplementary Figure S22.** Bud germination of rhizomes at 70 d.

**Supplementary Table S1.** Sample information.

**Supplementary Table S2.** Number of preferentially expressed genes in different organs.

**Supplementary Table S3.** GO enrichment analysis of genes preferentially expressed in different organs.

**Supplementary Table S4.** KEGG enrichment analysis of genes preferentially expressed in different organs.

**Supplementary Table S5.** The genes specifically expressed in different organs.

**Supplementary Table S6.** The mean expression of genes in different samples.

**Supplementary Table S7.** The TFs in 2 haplotypes.

**Supplementary Table S8.** Number of TFs preferentially expressed in different organs.

**Supplementary Table S9.** Mean values of the number of different *cis*-acting elements in the 2-kb promoter region of preferentially expressed genes in different organs.

**Supplementary Table S10.** The hub genes in the salmon module associated with ABRE *cis*-acting elements identified by WGCNA.

**Supplementary Table S11.** The expression of 16 TFs identified in the hub genes of the salmon module.

**Supplementary Table S12.** KEGG enrichment analysis of genes differentially expressed during rhizome development.

**Supplementary Table S13.** The expression of *P450* superfamily genes.

**Supplementary Table S14.** Distribution of ABA-related *cis*-acting elements predicted by PLACE and plantCARE in the 2-kb promoter region of candidate *P450* superfamily genes.

**Supplementary Table S15.** The clustering of gene expression patterns during the rhizome-to-bud transition.

**Supplementary Table S16.** TAU index of genes preferentially expressed in the rhizome and bud in Cluster 4.

**Supplementary Table S17.** GO enrichment analysis of genes preferentially expressed in the rhizome and bud in Cluster 4.

**Supplementary Table S18.** KEGG enrichment analysis of genes preferentially expressed in the rhizome and bud in Cluster 4.

**Supplementary Table S19.** The RT-qPCR primers for transgenic lines.

**Supplementary Table S20.** The primers used for the vector construction of candidate genes related to rhizome dormancy release.

**Supplementary Table S21.**  *Arabidopsis* seed germination under different ABA treatments.

**Supplementary Table S22.** The FPKM of key genes in the ABA and GA pathways associated with rhizome dormancy release.

**Supplementary Table S23.** Release of rhizome dormancy.

**Supplementary Table S24.** Effects of different treatments on rhizome dormancy release.

**Supplementary Table S25.** The RT-qPCR primers for candidate genes related to rhizome dormancy release.

**Supplementary Table S26.** TFs associated with rhizome dormancy release.

**Supplementary Table S27.** The Log_2_FPKM of candidate TFs that bind to the *CYP707A1* promoter.
