# Supplementary material for: The transcriptional landscape and dynamics regulating organ differentiation and dormancy in Curcuma alismatifolia
Source: Plant Physiol. 2025 Oct 13;199(2):kiaf501. doi: 10.1093/plphys/kiaf501 (PMC12569765; doi:10.1093/plphys/kiaf501)
Supplement: kiaf501_Supplementary_Data [file kiaf501_supplementary_data.zip › Supplementary Data.pdf]

Supplementary data

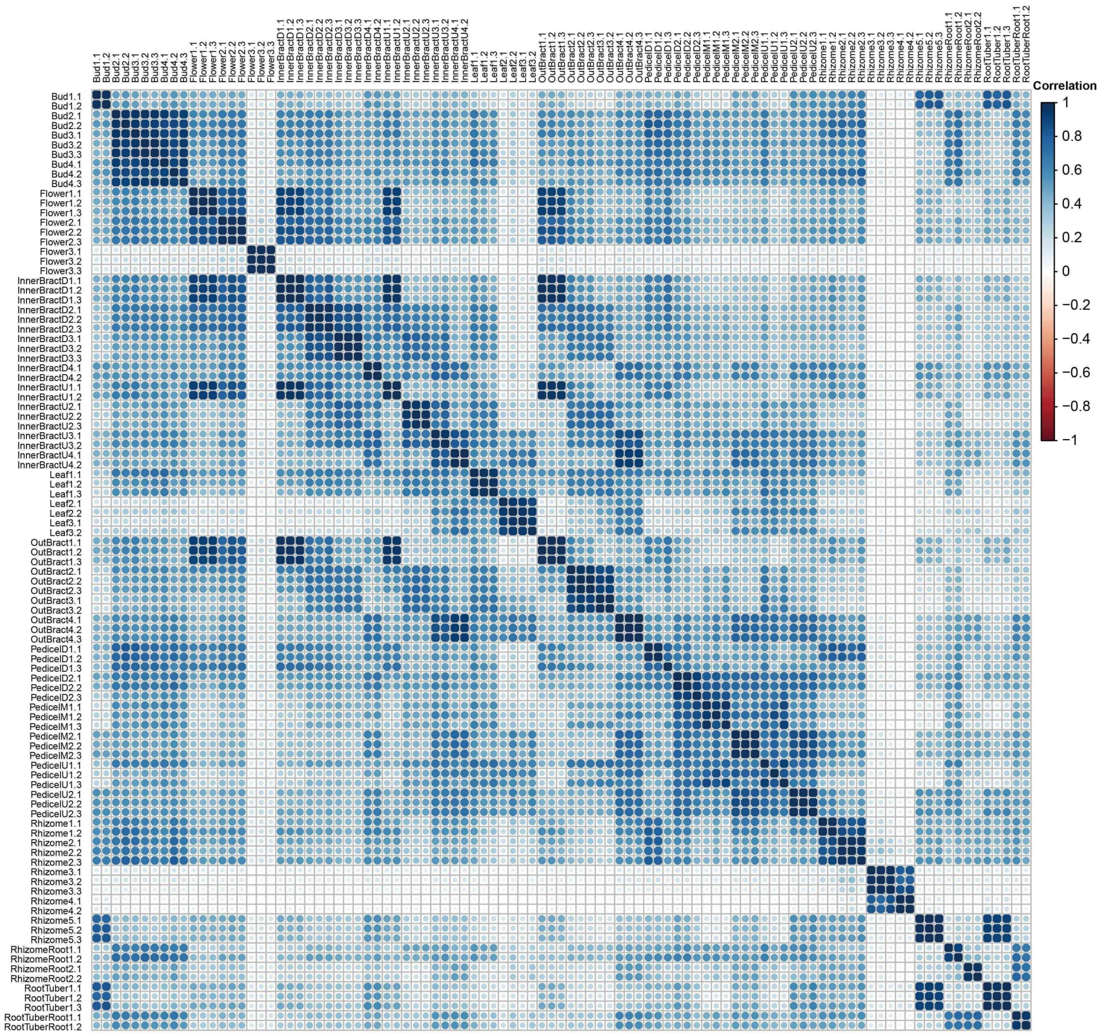

Supplementary Figure S1. Sample correlation analysis of *C. alismatifolia* RNA-seq data. Complete sample metadata are provided in Supplementary Table S1. D/M/U represent the base, middle, and tip of the sampling organ, respectively.

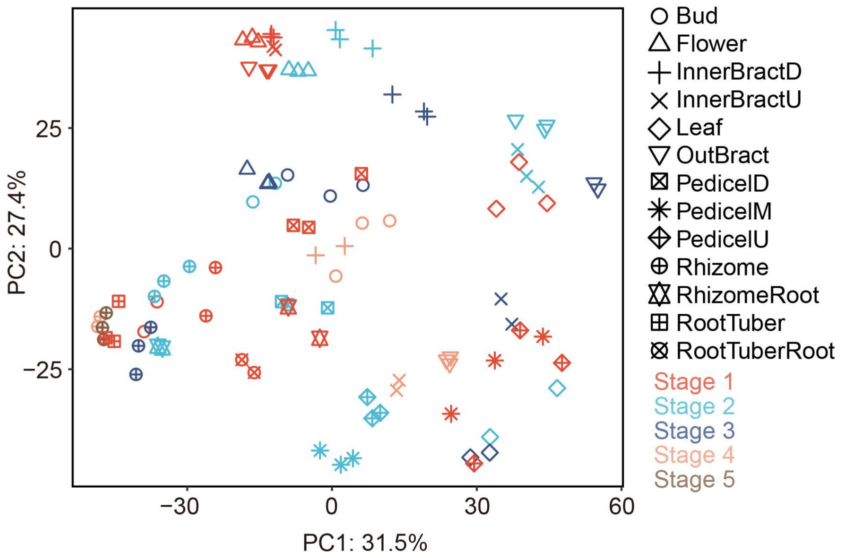

Supplementary Figure S2. PCA analysis of RNA-seq data derived from all 13 organ types across distinct developmental stages. Complete sample metadata are provided in Supplementary Table S1. D/M/U represent the base, middle, and tip of the sampling organ, respectively.

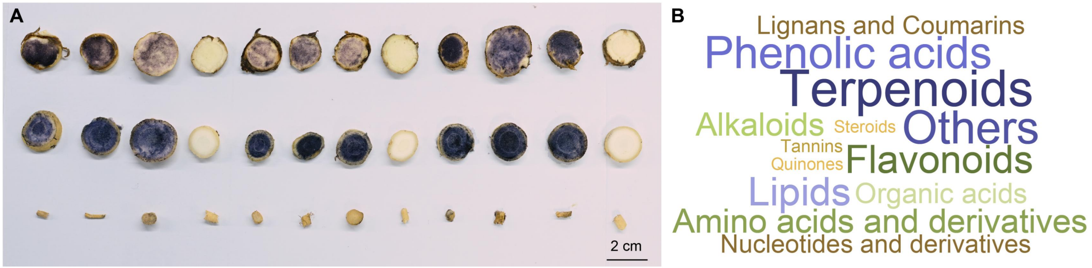

Supplementary Figure S3. Compounds from the *C. alismatifolia* rhizome. **A**) Iodine reaction of *C. alismatifolia* cv. 'Laddawan' (left), 'YuKi' (middle) and 'Chiang Mai Pink' (right) rhizomes (top), roots (middle) and tuberous roots (bottom), 3 individuals per cultivar tested. **B**) Distribution of secondary metabolites in rhizome4 of *C. alismatifolia*, with text font size indicating the amount of metabolite type.

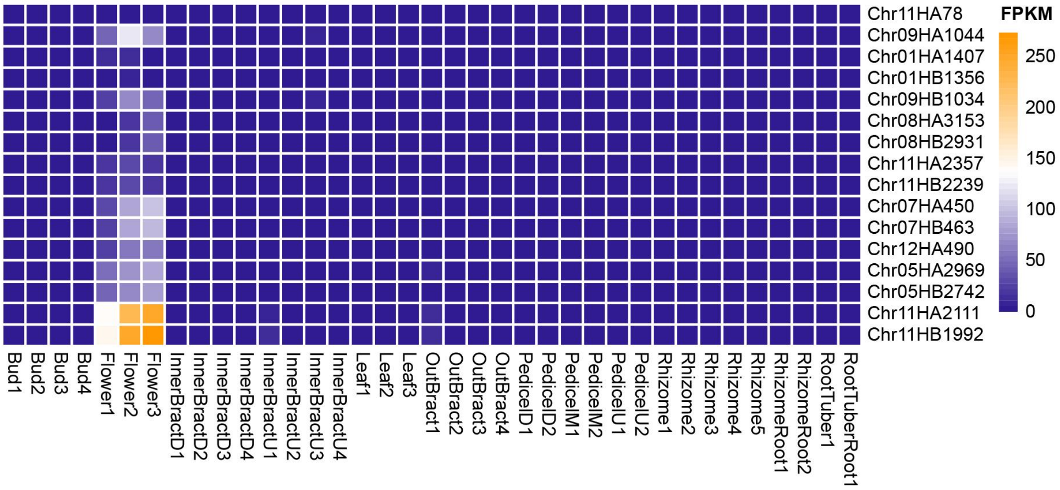

Supplementary Figure S4. MADS box transcription factors specifically expressed in flowers. The heatmap displays the expression patterns of MADS-box transcription factors with stronger flower specificity across different samples, identified using a stricter threshold (TAU ⩾ 0.99). D/M/U represent the base, middle, and tip of the sampling organ, respectively.

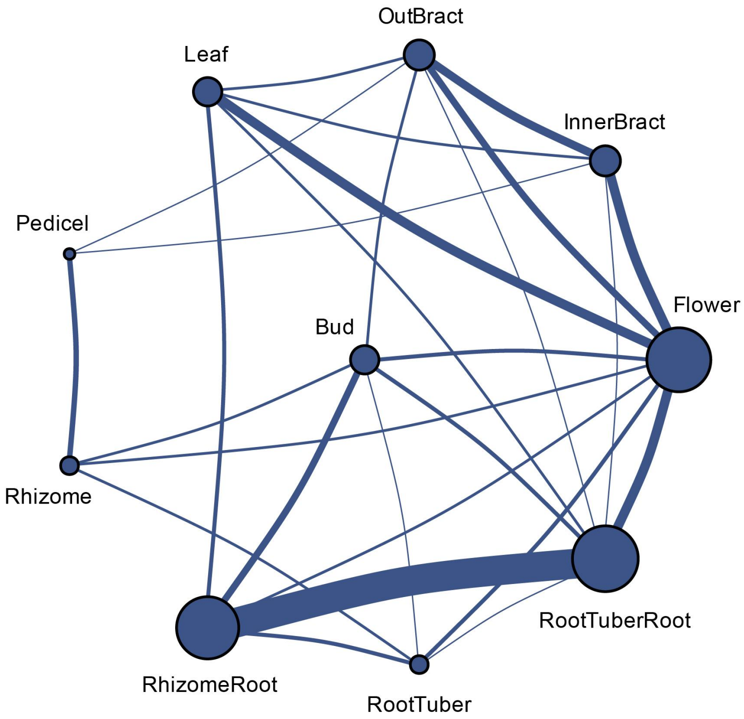

Supplementary Figure S5. Colinear gene pairs with different preferred samples. Thicker lines and larger circles indicate more genes.

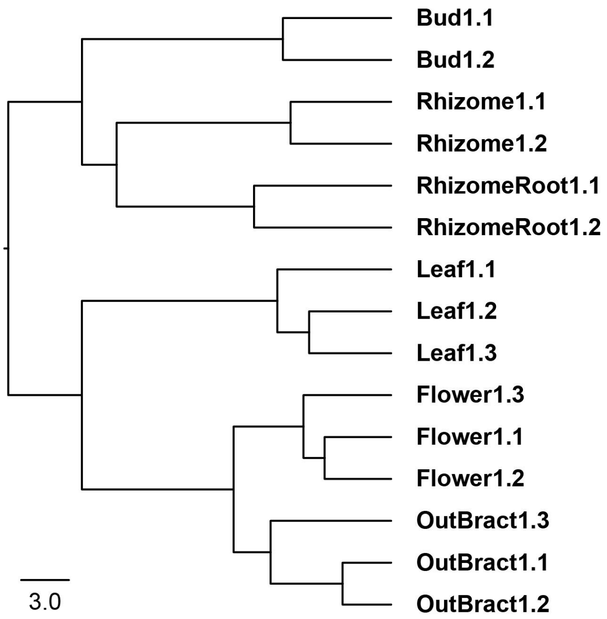

Supplementary Figure S6. Clustering of early samples across organizations. Sample clustering based on hierarchical clustering algorithms illustrates the distance between gene expression data across different samples. Samples located on the same branch exhibit higher similarity. The bar represents height, serving as a measure of similarity between samples.

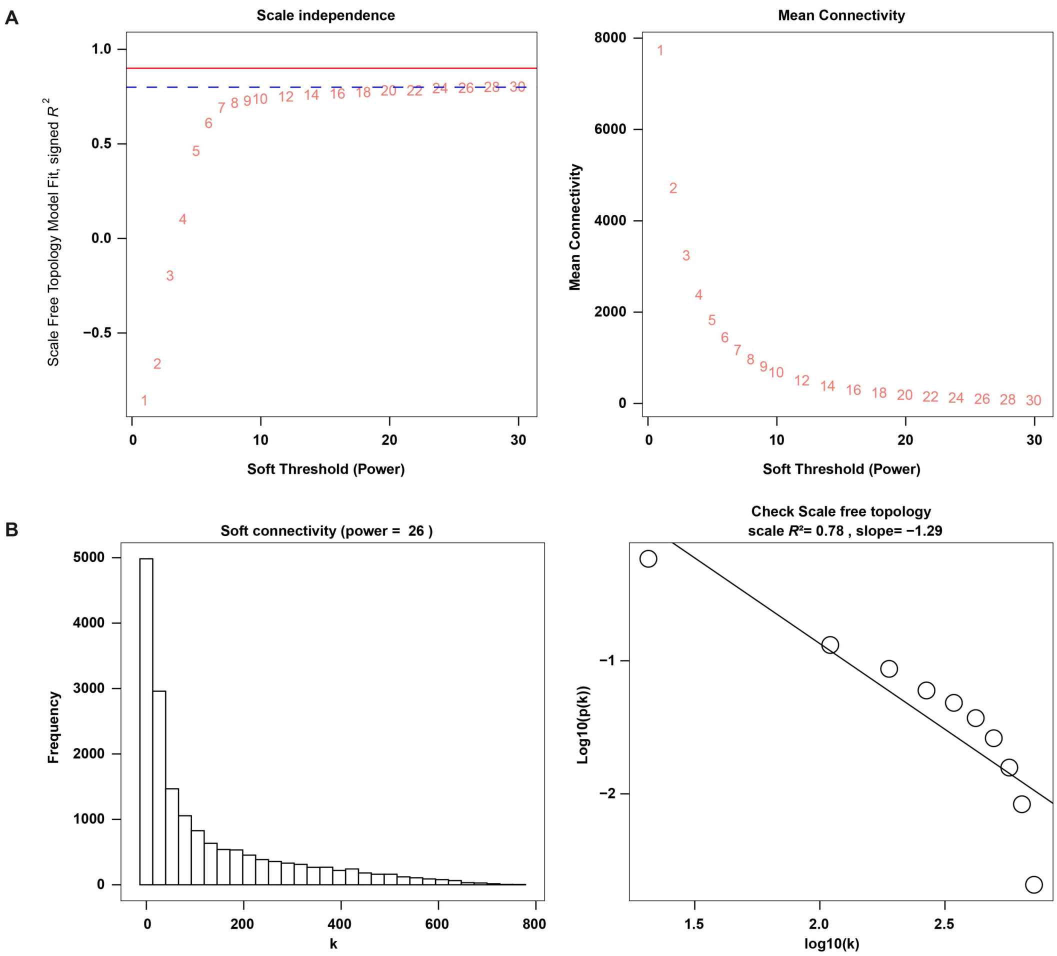

Supplementary Figure S7. Analysis of network topology for various soft-thresholding powers. **A**) Selection of the soft-thresholding powers. The left panel displays the scale-free index versus soft-thresholding power. The right panel shows the mean connectivity versus soft-thresholding power. The *x*-axis represents weighting parameters (power). The *y*-axis represents the scale-free fit index and connectivity for each power. **B**) Histogram of the number of node connections and validation that the network conforms to a scale-free distribution at a given threshold.

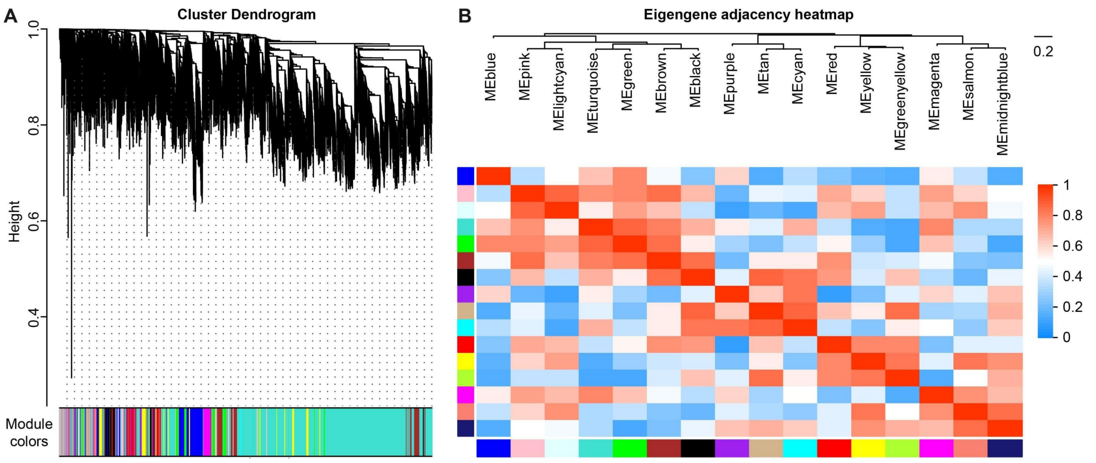

Supplementary Figure S8. Module detection by WGCNA. **A**) Module division. The upper panel shows the gene clustering, where branch height indicates distance and lower height reflects higher similarity among genes. The bottom panel shows modules, with nearby genes grouped into the same module. Each color represents one module, and gray denotes unassigned genes. **B**) Relationships between modules. Each color represents a module in the co-expression network by WGCNA. The upper panel shows hierarchical clustering of module eigengenes (MEs), where bar represents height and indicates similarity. The bottom panel shows the module correlation heatmap, with deeper red indicating stronger correlation.

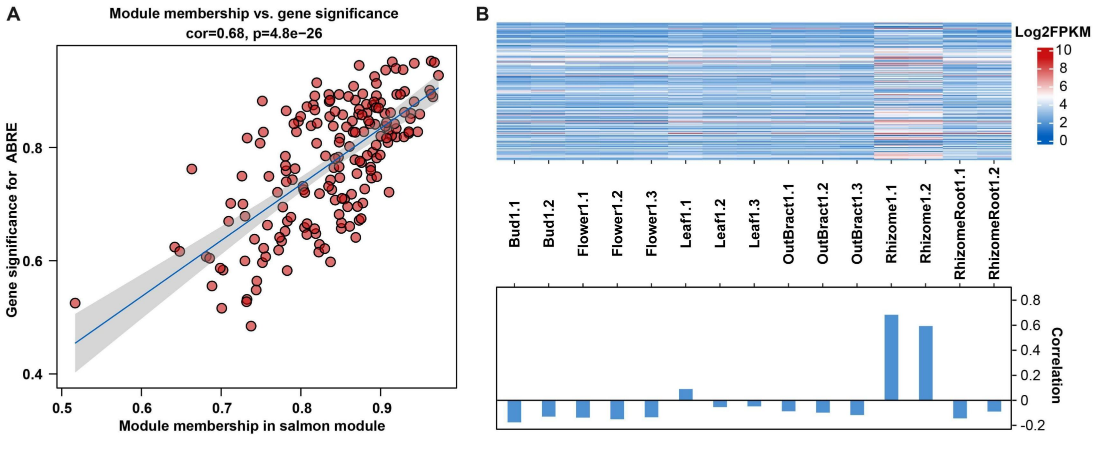

Supplementary Figure S9. Gene expression of salmon module associated with ABRE *cis*-elements. **A**) Scatterplot showing the correlation between gene significance and module membership in the salmon module. Genes closer to the top right show stronger module association and represent hub genes within the module. **B**) Expression of genes in the module. A heatmap of expression levels (Log2FPKM) of all genes in the salmon module across samples. Columns represent samples, rows represent genes in the salmon module. The bottom panel shows the correlation between the MEs and samples.

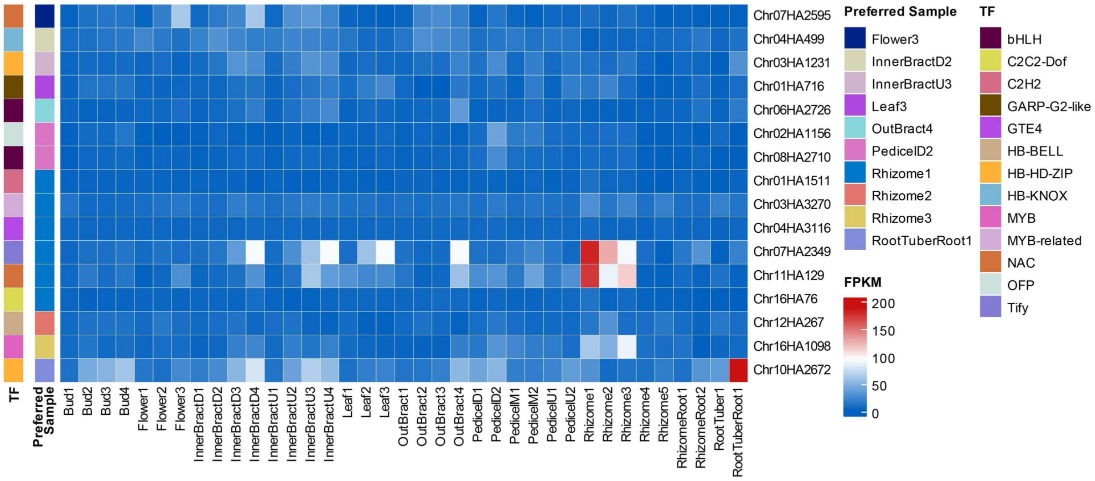

Supplementary Figure S10. Expression of TFs in hub genes within the salmon module and preferentially expressed organs. The *x*-axis indicates different samples, while the *y*-axis indicates the samples with preferential expression of different genes. Classes of different transcription factors are distinguished by different colors.

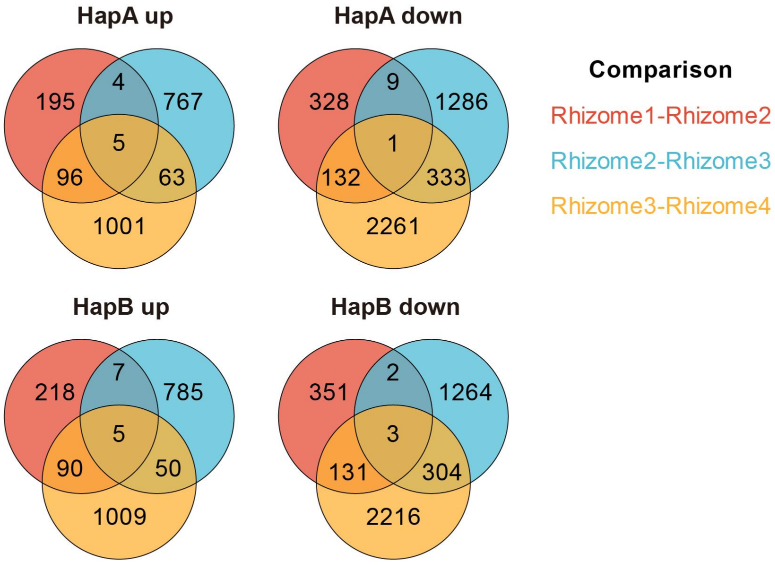

Supplementary Figure S11. Number of differentially expressed genes in different haplotypes during rhizome development. up, number of up-regulated expressed genes; down, number of down-regulated expressed genes. HapA, haplotype A; HapB, haplotype B.

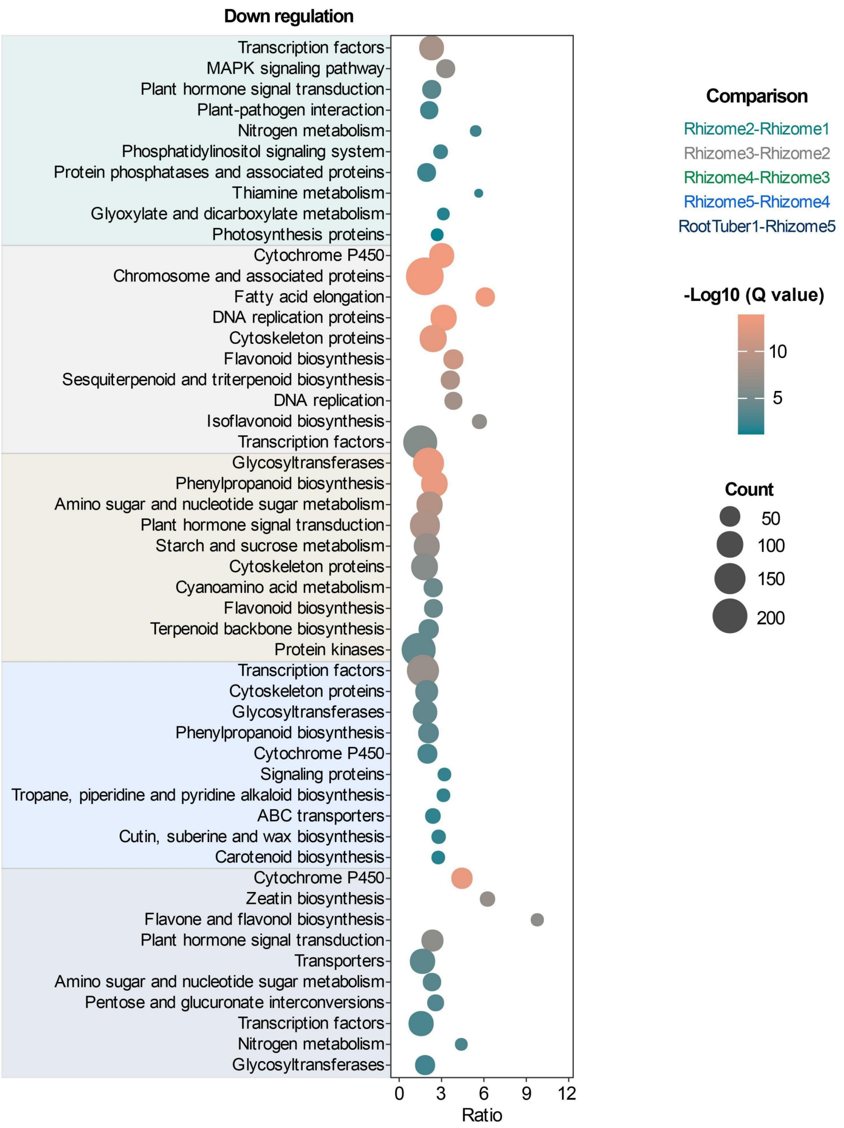

Supplementary Figure S12. KEGG enrichment analysis of the down-regulated differentially expressed genes in haplotype B during rhizome development and between rhizomes and tuberous roots in *C. alismatifolia*. Comparisons between samples at different rhizome developmental stages, as well as between tuberous root and rhizome at bud emergence stage, are represented by differently colored squares. The size of the circles indicates the enrichment level of genes across different pathways.

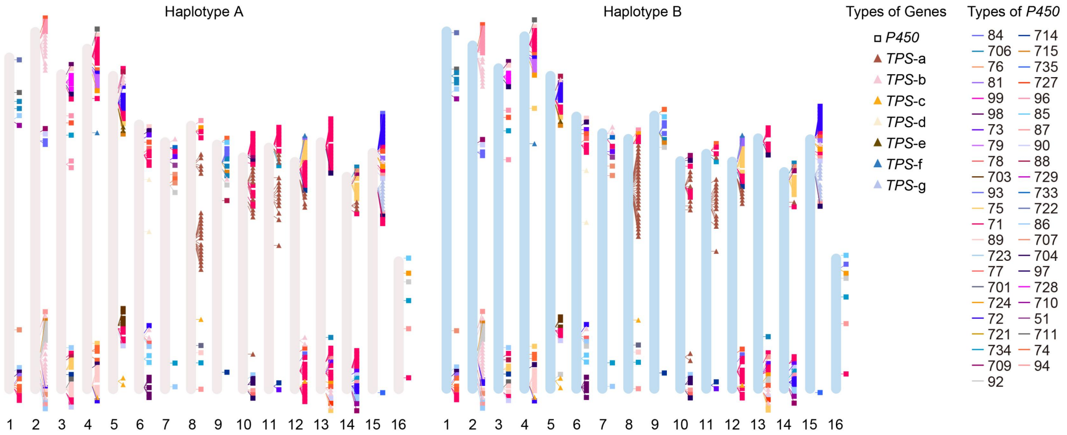

Supplementary Figure S13. Distribution of *P450* superfamily and terpene synthase (*TPS*) genes in both haplotypes of *C. alismatifolia*. Squares represent *P450* genes, while triangles denote *TPS* genes. Different colors represent different classifications of *P450*/*TPS* genes.

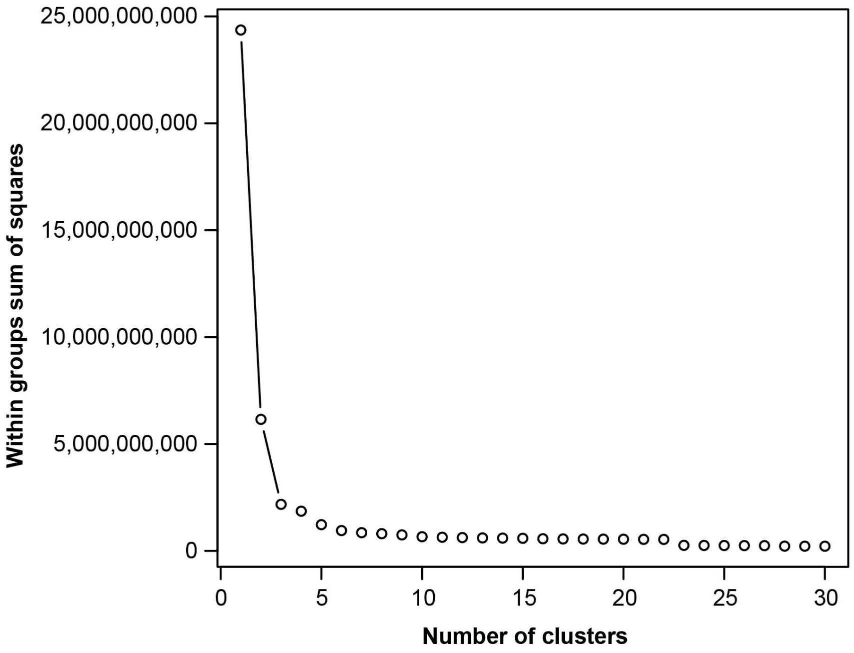

Supplementary Figure S14. Determination of the best cluster number for k-means. By plotting the relationship between the sum of squared errors (SSE) within clusters and the K value, it can be observed that as K increases, SSE gradually decreases. The inflection point where the rate of SSE decline begins to slow represents the optimal K value. This K value (K = 5) was subsequently employed for cluster analysis in the Mfuzz software.

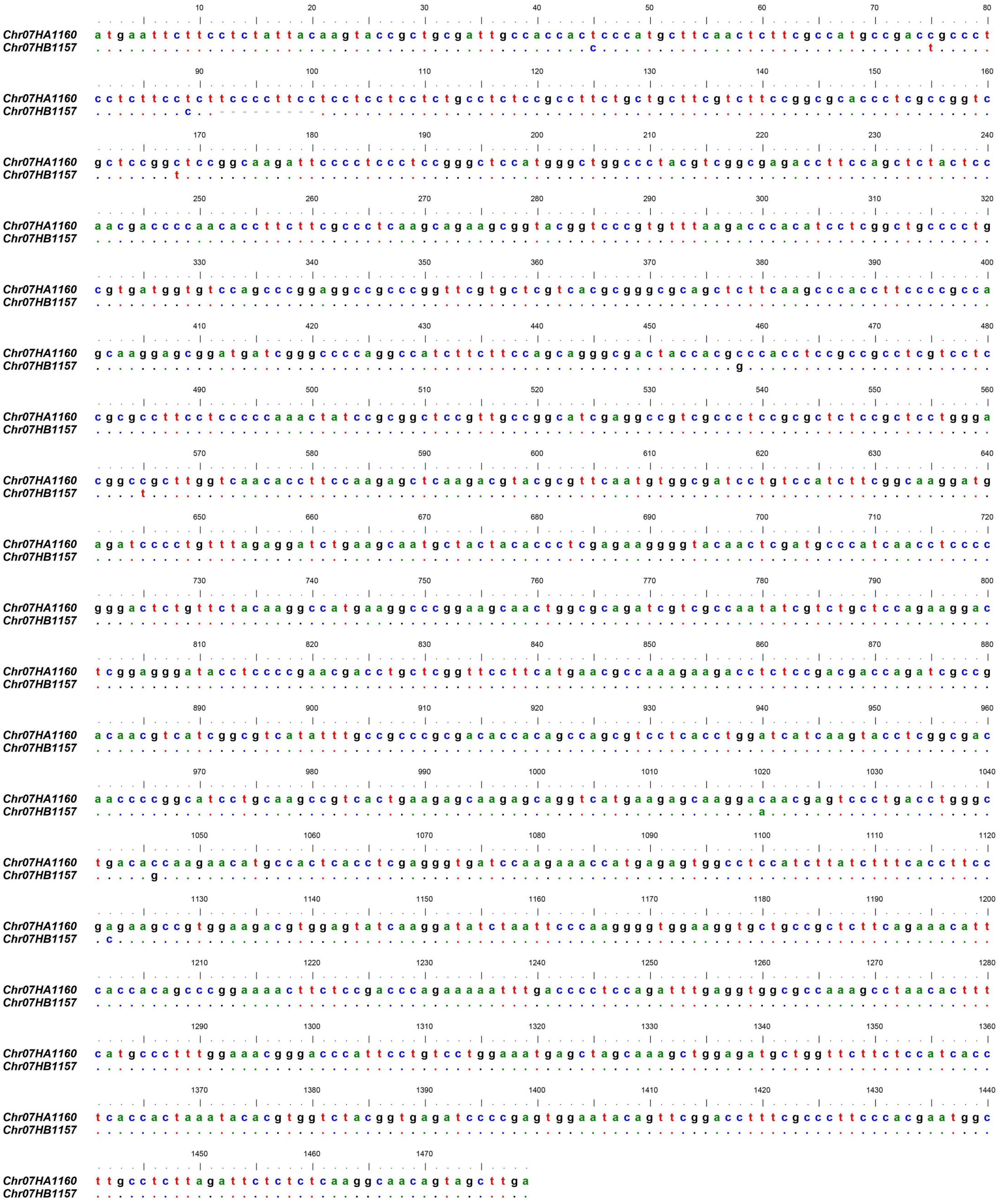

Supplementary Figure S15. CDS alignment of the *CYP707A1* gene *Chr07HA1160/Chr07HB1157*. The CDS alignment of the *CYP707A1* gene located on two haplotypes of *C. alismatifolia* (i.e., *Chr07HA1160* and *Chr07HB1157*) is shown, with different bases indicated by different colors.

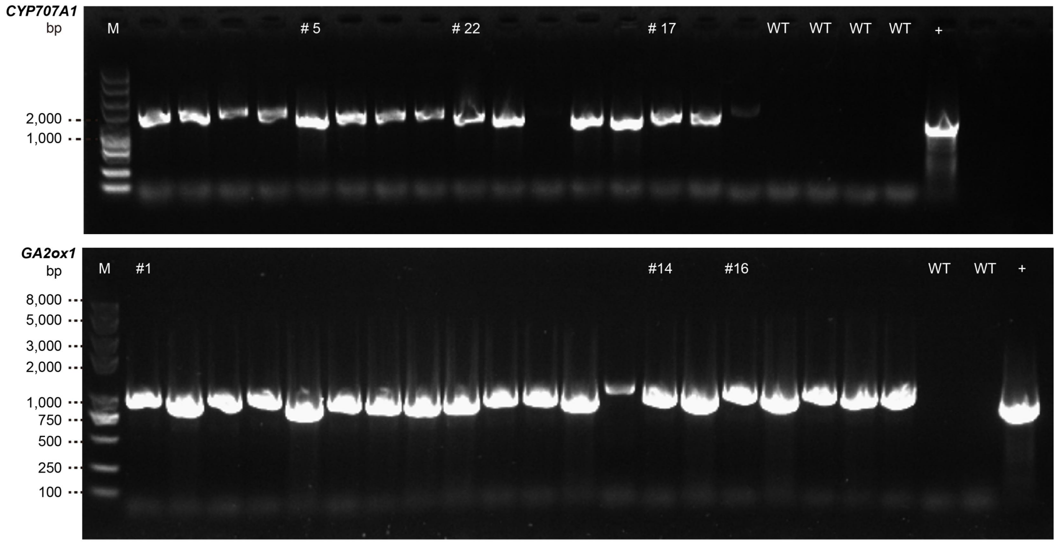

Supplementary Figure S16. PCR validation of transgenic *A. thaliana* lines. The upper panel shows PCR validation of *CYP707A1* (*Chr07HB1157*) transgenic *A. thaliana* lines. The bottom panel shows PCR validation of *GA2ox1* (*Chr11HA2367*) transgenic *A. thaliana* lines. M: marker, #: positive lines used for subsequent analysis, +: positive control, WT: wild type.

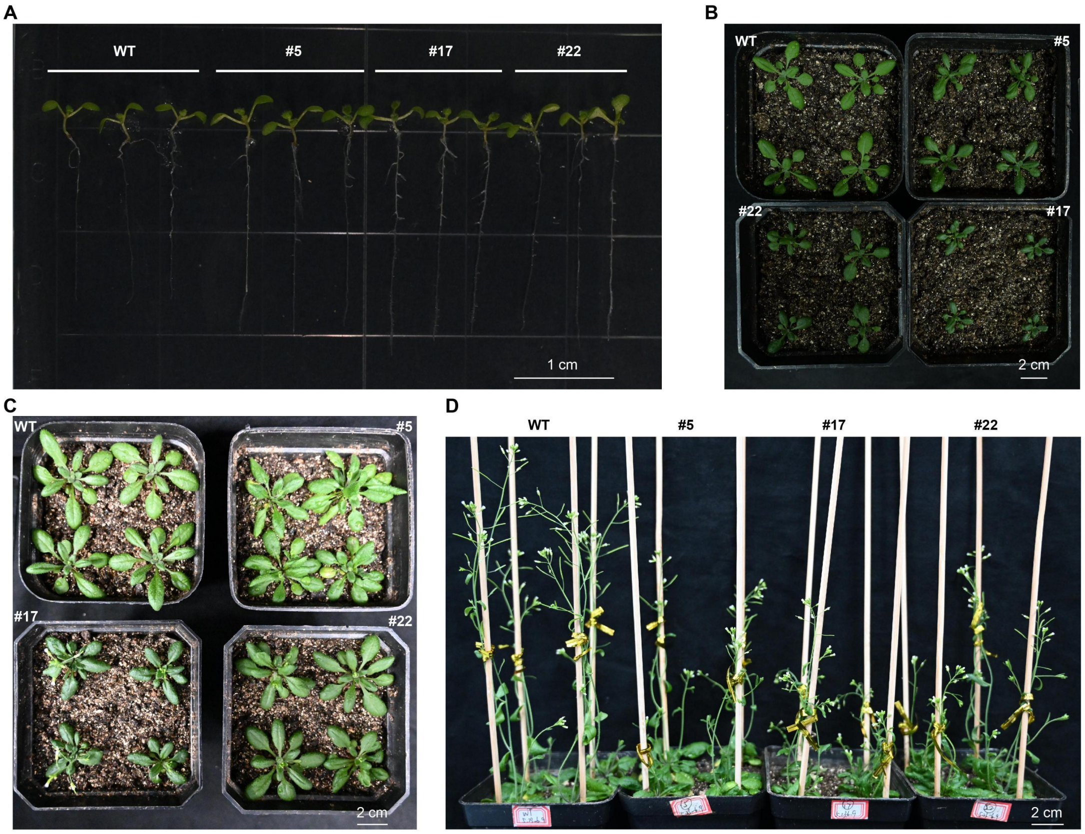

Supplementary Figure S17. Phenotypic characterization of *CYP707A1*-overexpressing *A. thaliana* plants. **A)** Root length at 10 days post-germination. **B)** The phenotype of rosettes at bolting initiation in transgenic plants. One plant image from each line is identical to the sample in Figure 4G. **C)** The phenotype of rosettes at bolting initiation in wild-type plants. **D)** Plant height at 50% flowering. Bar = 1 cm. WT: wild-type *Col-0*; #5, #17, #22: independent *CYP707A1* transgenic T2 lines.

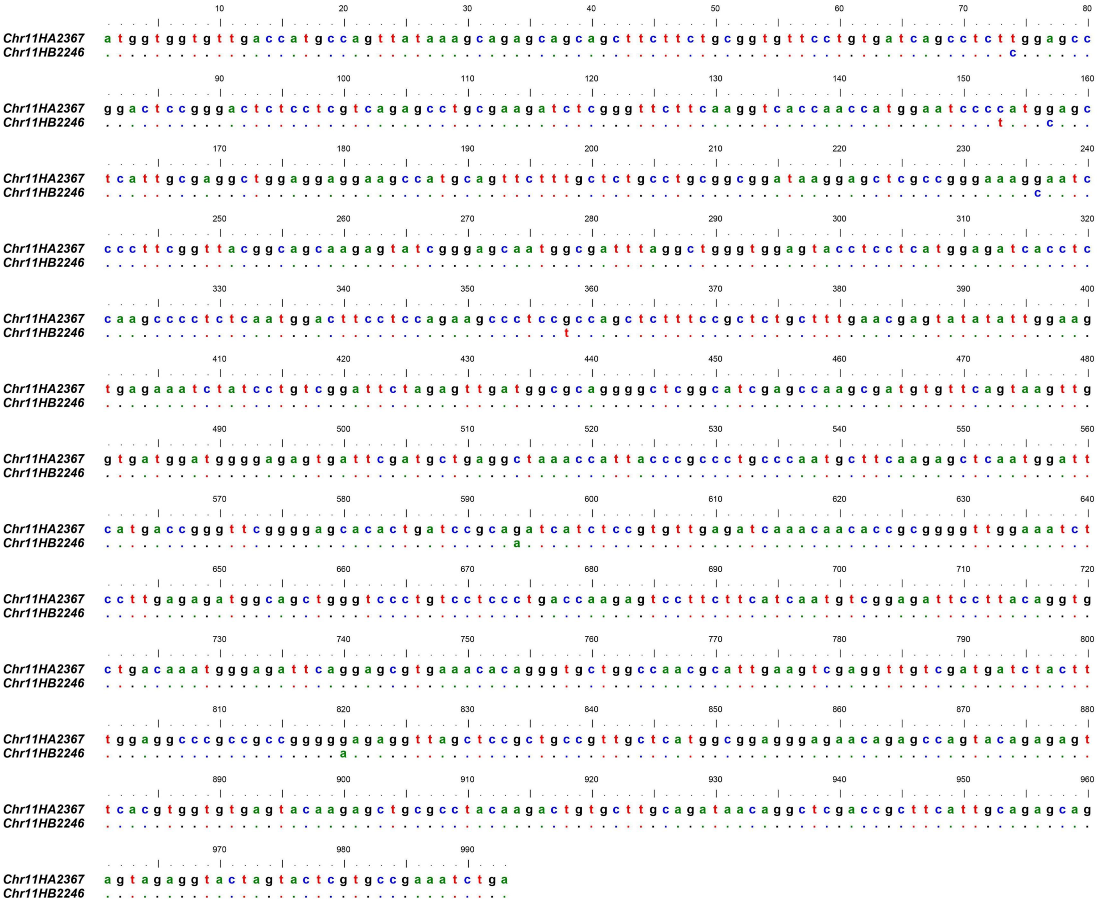

Supplementary Figure S18. CDS alignment of the *GA2ox1* gene *Chr11HA2367/Chr11HB2246*. The CDS alignment of the *GA2ox1* gene located on two haplotypes (i.e., *Chr11HA2367* and *Chr11HB2246*) is shown, with different bases indicated by different colors.

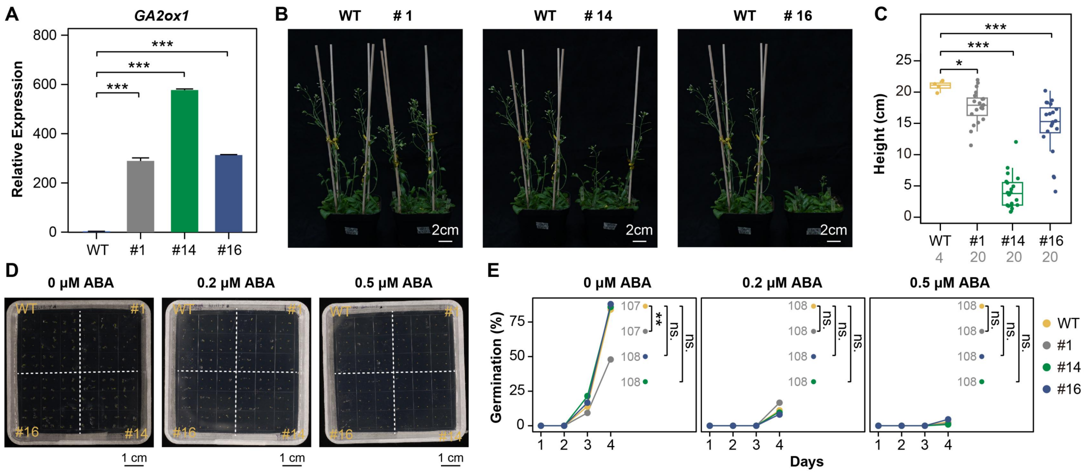

Supplementary Figure S19. The T2 generation of *GA2ox*-overexpressing transgenic *Arabidopsis thaliana* and its phenotypes. **A**) Validation of RT-qPCR for T2 lines of *GA2ox* transgenic *A. thaliana*. Results are reported as mean ± standard deviation (SD). $n = 3$. Statistical tests were two-sided Student's *t*-test, and multiple comparisons were adjusted with the Bonferroni correction. Asterisks represented significant differences (***$p < 0.001$, adjusted). **B**) The phenotype of transgenic T2 lines. The wild-type plants used in each comparison are identical. **C**) Plant height at 50% flowering. The box-plot elements were defined as: center line, median; box limits, upper and lower quartiles; whiskers, 1.5× interquartile range; points, all values. Two-sided Wilcoxon rank-sum test was conducted for significance evaluation, and multiple comparisons were adjusted with the Bonferroni correction. Asterisks represented significant differences (*$p < 0.05$, ***$p < 0.001$, adjusted). The gray numbers indicate the sample size. **D**) Seed germination of transgenic T2 lines on medium containing different concentrations of ABA. **E**) Seed germination rate of transgenic T2 lines. $n = 3$. Statistical tests were two-sided Student's *t*-test, and multiple comparisons were adjusted with the Bonferroni correction. Asterisks represented significant differences (**$p < 0.01$, adjusted) (Supplementary Table S21). The gray numbers indicate the sample size.

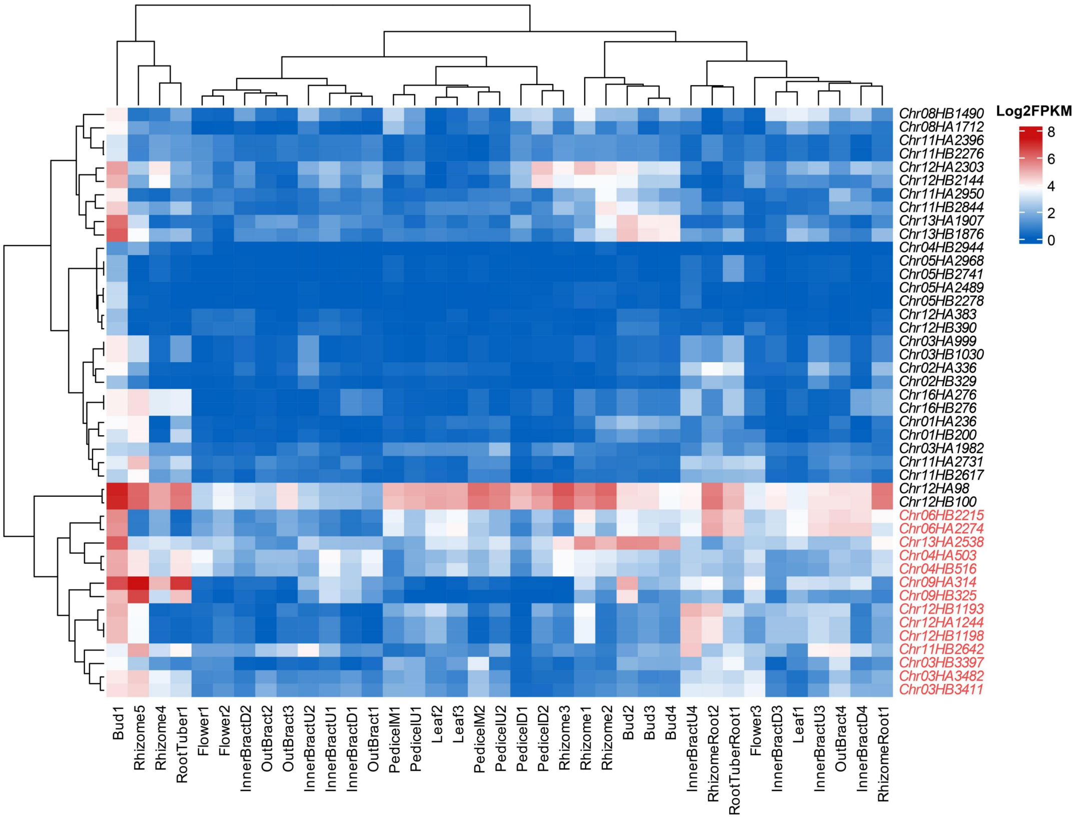

Supplementary Figure S20. Expression clustering of candidate transcription factors (TFs) that regulate *CYP707A1*. Heatmap displays Log2FPKM transformed expression of TFs after filtering: 1) Removal of TFs with FPKM ≤ 2 in any sample; 2) Exclusion of TFs lacking predicted binding sites in the *CYP707A1* promoter (verified using JASPAR (https://jaspar.elixir.no/) and PlantPAN 4.0 (https://plantpan.itps.ncku.edu.tw/plantpan4/promoter_analysis.php)). Genes marked in red are final candidates.

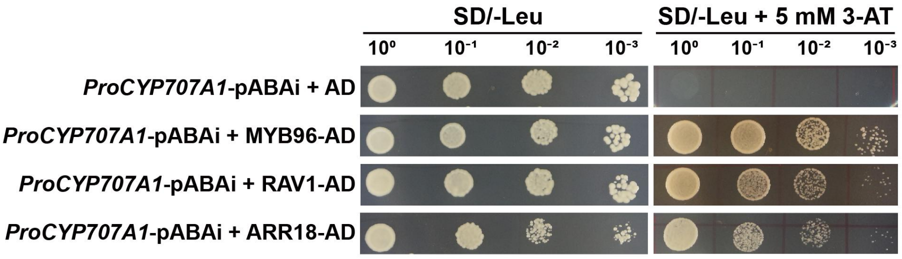

Supplementary Figure S21. Y1H assay showing that MYB96, RAV1 and ARR18 bind to the *CYP707A1* promoter in yeast cells.

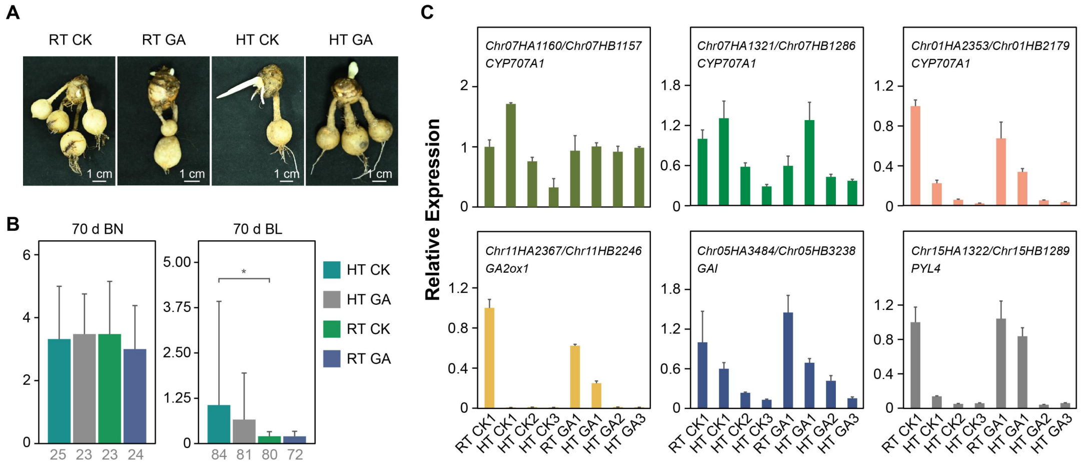

Supplementary Figure S22. Bud germination of rhizomes at 70 d. **A**) Phenotype of rhizomes at 70 d. The RT CK is the same as the RT in Figure 5B, and the HT CK is the same as the HT in Figure 5B. **B**) Number of bud and bud length of 70 d rhizomes. HT CK: 30°C and no 20 mg/L GAs treatment, HT GA: 30°C and 20 mg/L GAs treatment, RT CK: 22°C and no 20 mg/L GAs treatment, RT GA: 22°C and 20 mg/L GAs treatment. Results are reported as mean ± standard deviation (SD). Two-sided Wilcoxon rank-sum test was conducted for significance evaluation, and multiple comparisons were adjusted with the Bonferroni correction. Asterisks represented significant differences (**$p < 0.05$, adjusted) (Supplementary Table S20 and S21). The gray numbers indicate the sample size. **C**) Expression of candidate genes in rhizomes at 70 d based on RT-qPCR. $n = 3$. RT CK1: ungerminated buds in ambient conditions (22 °C), HT CK1: ungerminated buds in high temperature conditions (30 °C), HT CK2: 0.1-0.5 cm buds under high temperature conditions (30 °C), HT CK3: 2-3 cm buds under high temperature conditions (30 °C). RT GA1: ungerminated buds with 20 mg/L GAs treatment in ambient conditions (22 °C), HT GA1: ungerminated buds with 20 mg/L GAs treatment in high temperature conditions (30 °C), HT GA2: 0.1-0.5 cm buds under high temperature conditions (30 °C) with 20 mg/L GAs treatment, HT GA3: 2-3 cm buds under high temperature conditions (30 °C) with 20 mg/L GAs treatment. Results are reported as mean ± standard deviation (SD).
